# Identification of new correctors for traffic-defective ABCB4 variants by a high-content screening approach

**DOI:** 10.1038/s42003-024-06590-y

**Published:** 2024-07-24

**Authors:** Mounia Lakli, Julie Dumont, Virginie Vauthier, Julie Charton, Veronica Crespi, Manon Banet, Yosra Riahi, Amel Ben Saad, Elodie Mareux, Martine Lapalus, Emmanuel Gonzales, Emmanuel Jacquemin, Florent Di Meo, Benoit Deprez, Florence Leroux, Thomas Falguières

**Affiliations:** 1 Inserm, Université Paris-Saclay, Physiopathogénèse et traitement des maladies du foie, UMR_S 1193, Hepatinov, F-91400 Orsay, France; 2 Université de Lille, Inserm, Institut Pasteur de Lille, U1177 – Drugs and Molecules for Living Systems, F-59000 Lille, France; 3 Université de Lille, CNRS, Inserm, CHU Lille, Institut Pasteur de Lille, US 41 - UAR 2014 - PLBS, F-59000 Lille, France; 4 Inserm, Sorbonne Université, Centre de Recherche Saint-Antoine (CRSA), UMR_S 938, Institute of Cardiometabolism and Nutrition (ICAN), F-75012 Paris, France; 5 Inserm, Université de Limoges, Pharmacology & Transplantation, UMR 1248, Centre de Biologie et Recherche en Santé, F-87000 Limoges, France; 6 Assistance Publique - Hôpitaux de Paris, Paediatric Hepatology & Paediatric Liver Transplant Department, Reference Center for Rare Paediatric Liver Diseases, FILFOIE, ERN RARE LIVER, Faculté de Médecine Paris-Saclay, CHU Bicêtre, F-94270 Le Kremlin-Bicêtre, France

**Keywords:** High-throughput screening, Protein transport, Experimental models of disease, Molecular modelling, Mechanisms of disease

## Abstract

ABCB4 is located at the canalicular membrane of hepatocytes and is responsible for the secretion of phosphatidylcholine into bile. Genetic variations of this transporter are correlated with rare cholestatic liver diseases, the most severe being progressive familial intrahepatic cholestasis type 3 (PFIC3). PFIC3 patients most often require liver transplantation. In this context of unmet medical need, we developed a high-content screening approach to identify small molecules able to correct ABCB4 molecular defects. Intracellularly-retained variants of ABCB4 were expressed in cell models and their maturation, cellular localization and function were analyzed after treatment with the molecules identified by high-content screening. In total, six hits were identified by high-content screening. Three of them were able to correct the maturation and canalicular localization of two distinct intracellularly-retained ABCB4 variants; one molecule was able to significantly restore the function of two ABCB4 variants. In addition, in silico molecular docking calculations suggest that the identified hits may interact with wild type ABCB4 residues involved in ATP binding/hydrolysis. Our results pave the way for their optimization in order to provide new drug candidates as potential alternative to liver transplantation for patients with severe forms of ABCB4-related diseases, including PFIC3.

## Abstract (web summary)

A high-content screening using a fluorescence readout has been used to identify pharmacological correctors of intracellularly-retained ABCB4 variants. The identified small molecules rescued canalicular targeting of ABCB4 variants.

## Introduction

The ABCB4 transporter plays an essential role in bile secretion^1^. This transmembrane glycoprotein is a member of the superfamily of ATP-binding cassette (ABC) transporters, that harvest energy from ATP binding/hydrolysis for translocating a wide variety of substrates across biological membranes^2,3^. ABCB4 is a floppase exclusively expressed by hepatocytes at the canalicular membrane^4^ where it flops phosphatidylcholine (PC) from the inner to the outer leaflet of this membrane^5–7^. Through the formation of mixed micelles, ABCB4 contributes to solubilize cholesterol, preventing the formation of gallstones, and protect the biliary epithelium from the detergent action of free bile salts^8,9^. Mutations in the *ABCB4* gene are associated with several cholestatic diseases, the most severe form being progressive familial intrahepatic cholestasis type 3 (PFIC3)^10,11^. This rare autosomal recessive disease generally leads to clinical signs of cholestasis and cirrhosis during the first years of life. Liver transplantation is required before adulthood in half of PFIC3 patients^10,12,13^. Ursodeoxycholic acid, which is the current standard treatment for ABCB4-related diseases, only improves approximately 50% of PFIC3 patients^10,14,15^. This important unmet medical need stresses the identification of new treatments.

To date, more than 500 genetic variations of the *ABCB4* locus have been reported from patients (https://gnomad.broadinstitute.org/; https://bravo.sph.umich.edu/; http://abcm2.hegelab.org/ - accessed on 22/01/2024), most of them being missense mutations and affecting single individuals/families (private mutations). These variations can impact ABCB4 protein at several levels: expression (class I), maturation and exit from the endoplasmic reticulum (ER) (class II), PC secretory function (class III), or plasma membrane stability (class IV)^16,17^. This classification is of utmost importance to consider targeted pharmacotherapies adapted to the class of the variants carried by the patients in the frame of a personalized therapeutic strategy.

Focusing on traffic-defective ABCB4 variants (class II), many efforts have been made to develop new therapies in order to avoid or at least delay liver transplantation. The proportion represented by class II variants is hard to estimate since most of ABCB4 variants have not been characterized at the molecular and cellular levels. However, based on a former study investigating a small number of missense variants^16,17^, a rough estimation would be that class II variants represent 20 to 30% of total missense variants. In a repurposing strategy, we have previously shown that some correctors of the ER-retained F508del variant of ABCC7/CFTR (cystic fibrosis transmembrane conductance regulator) partially corrected the maturation and plasma membrane localization of class II ABCB4 variants^18^. However, these correctors have not been further investigated because of their inhibitory effect on ABCB4 function^18^. Other compounds derived from roscovitine have been shown to partially correct both traffic and function of selected ER-retained variants of ABCB4^19^. Repurposing strategies, although effective and easily transferrable to clinics, are limited to approved molecules, based on studies carried out in the frame of other pathologies, including those involving other ABC transporters, mostly ABCC7/CFTR.

We here performed high-content screening (HCS; for a review see ref. ^20^) of an important diversity of small molecules, including food and drug administration (FDA)-approved compounds present in the commercial Prestwick chemical library^®^, using the well-characterized I541F variant of ABCB4^12,21,22^ as a model of ER retention (class II). Six hits were identified, of which three have been further investigated in cell models for their efficacy in rescuing the maturation, plasma membrane localization, and residual function of three ER-retained ABCB4 variants (I541F, L556R and I490T). These three drug candidates corrected the maturation and canalicular localization of two ABCB4 variants (I541F and L556R) while two compounds at different doses allowed a function restoration for ABCB4-I541F and one compound allowed a function restoration for ABCB4-L556R. No significant correction was observed for ABCB4-I490T after drug treatment. To better understand the mode of action of these molecules, we investigated their possible direct interaction with wild type ABCB4 using in silico molecular docking calculations.

## Results

### Development of a high-content screening assay for identifying ABCB4 correctors

**Fig. 1:**
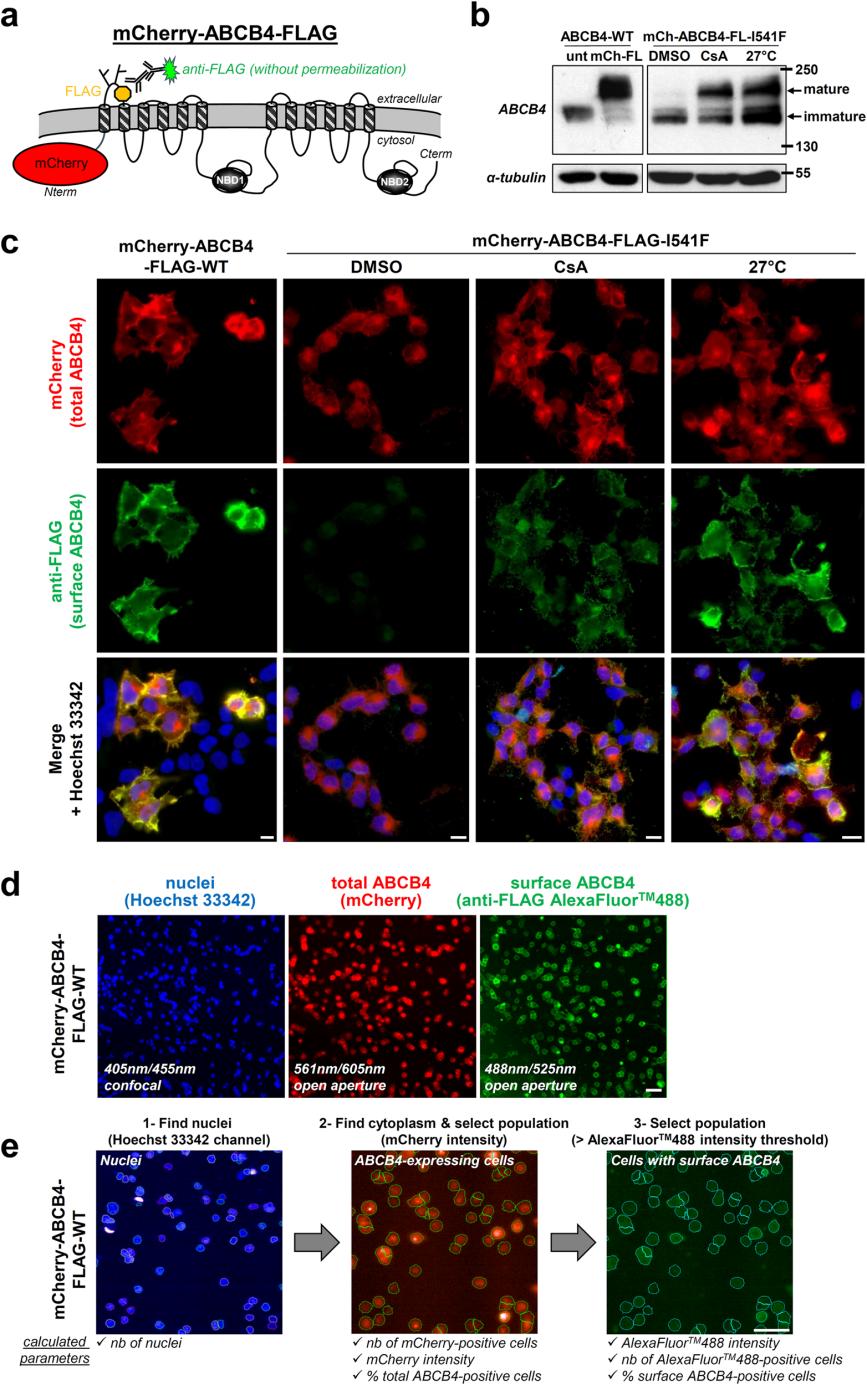
Development of a modified version of ABCB4 for HCS. **a** The membrane topology of the mCherry-ABCB4-FLAG construct is represented. NBD, nucleotide-binding domain. **b** ABCB4-WT without tag (unt) or double tagged (mCh-FL: mCherry-ABCB4-FLAG), or mCherry-ABCB4-FLAG-I541F (mCh-ABCB4-FL-I541F) were transiently expressed in HEK cells. After the indicated treatment (16 h with 10 µM CsA or at 27 °C), cell lysates were prepared and analyzed by immunoblot using the indicated antibodies. ABCB4-WT is shown as reference. The mature and immature forms of ABCB4 are indicated, as well as molecular weight markers. This panel is representative of three independent experiments. Full immunoblots are shown in Supplementary Fig. 14a. **c** mCherry-ABCB4-FLAG-WT or mCherry-ABCB4-FLAG-I541F were transiently expressed in HEK cells. Cells were treated 16 h as in (**b**), then fixed but not permeabilized. After anti-FLAG immunolabeling, total (red) *vs* plasma membrane (green) ABCB4 were visualized by confocal microscopy. Nuclei shown in the merged images were labeled with Hoechst 33342 (blue). This panel is representative of three independent experiments. Bar: 10 μm. **d** Same as (**c**), except that constructs were stably expressed in HEK cells, sample processing was miniaturized and image acquisition was performed using an automated confocal microscope. Then, images were batch analyzed using a script written with Columbus™ (e). Bar: 50 µm. **e** Software-assisted image analysis. After nuclei detection with Hoechst 33342 blue channel (1), ABCB4-expressing cells were selected with the mCherry red channel (2). The cell surface ABCB4 subpopulation was detected using anti-FLAG (and AlexaFluor^TM^488) labeling (3). The number of total, ABCB4-positive and surface ABCB4-expressing cells were determined; red and green signal intensities were measured. Bar: 50 µm.

In order to identify new correctors for traffic-defective ABCB4 variants, we developed a HCS approach with a fluorescence readout to discriminate between cell surface *vs* total ABCB4. First, a modified version of ABCB4 was built with a mCherry tag at its N-terminus and a FLAG tag inserted in its first extracellular loop (mCherry-ABCB4-FLAG-wild type; WT), enabling the detection of total *vs* cell surface transporters in non-permeabilized cells, respectively (Fig. 1a). The well-characterized class II I541F variant^16,21,23^ was used as a prototype of ER-retained ABCB4. mCherry-ABCB4-FLAG-I541F was expressed in HEK (human embryonic kidney) cells and, as expected, was mostly present on immunoblot as an immature protein due to its incomplete glycosylation as compared to its wild type counterpart (Fig. 1b)^21^. The maturation defect of mCherry-ABCB4-FLAG-I541F was partially corrected by treating cells with cyclosporin A (CsA) or by lowering cell culture temperature at 27 °C (Fig. 1b), as observed for untagged ABCB4-I541F^21,23^. Immunofluorescence analyses showed that mCherry-ABCB4-FLAG-WT was present at the plasma membrane, in contrast to the ER-retained I541F variant (Fig. 1c, green labeling). As suggested by immunoblot analysis (Fig. 1b), CsA treatment or cultivating cells at 27 °C corrected plasma membrane localization of mCherry-ABCB4-FLAG-I541F (Fig. 1c).

Aiming at miniaturizing and automatizing this approach for HCS, we stably expressed these constructs in HEK cells, which showed the best expression in amount and time as compared to other cell types. Since a direct access to the plasma membrane was required for the non-permeabilized system, hepatocyte-derived cell lines such as HepG2^24^ or Can 10^25^ are not suitable because their canalicular pole is not topologically accessible to antibodies without permeabilization. After stable expression of mCherry-ABCB4-FLAG-WT or -I541F in HEK cells, the approach was miniaturized in 384-well plates and the indirect immunofluorescence protocol was automatized. Fluorescence images were acquired using an automated confocal microscope (Fig. 1d and Supplementary Fig. 1) and well recapitulated our observations in non-miniaturized and non-automatized conditions (Fig. 1c). Images were further analyzed in silico using a customized script to generate statistically significant, quantitative and multi-parametric data in a fast and automated way (Fig. 1e; see details in Materials and Methods). Quantification analyses indicated that more than 95% of cells expressed the mCherry-tagged constructs in all conditions (Supplementary Fig. 2a). Although mCherry signal (total ABCB4) remained similar for the four tested conditions (Supplementary Fig. 2b), cell surface expression of ABCB4 was dramatically reduced for mCherry-ABCB4-FLAG-I541F (anti-FLAG signal intensity values of 20 ± 10 AU), as compared with its WT counterpart (140 ± 32 AU), and partially corrected after 10 µM CsA treatment (95 ± 35 AU) or low temperature cell culture (105 ± 23 AU), as expected (Supplementary Fig. 2c). Altogether, these results indicate that this double-tagged version of ABCB4 is a suitable tool to screen drug libraries by automated means using a fluorescence quantitative image-based readout.

### A first high-content screening using an FDA-approved chemical library

**Fig. 2:**
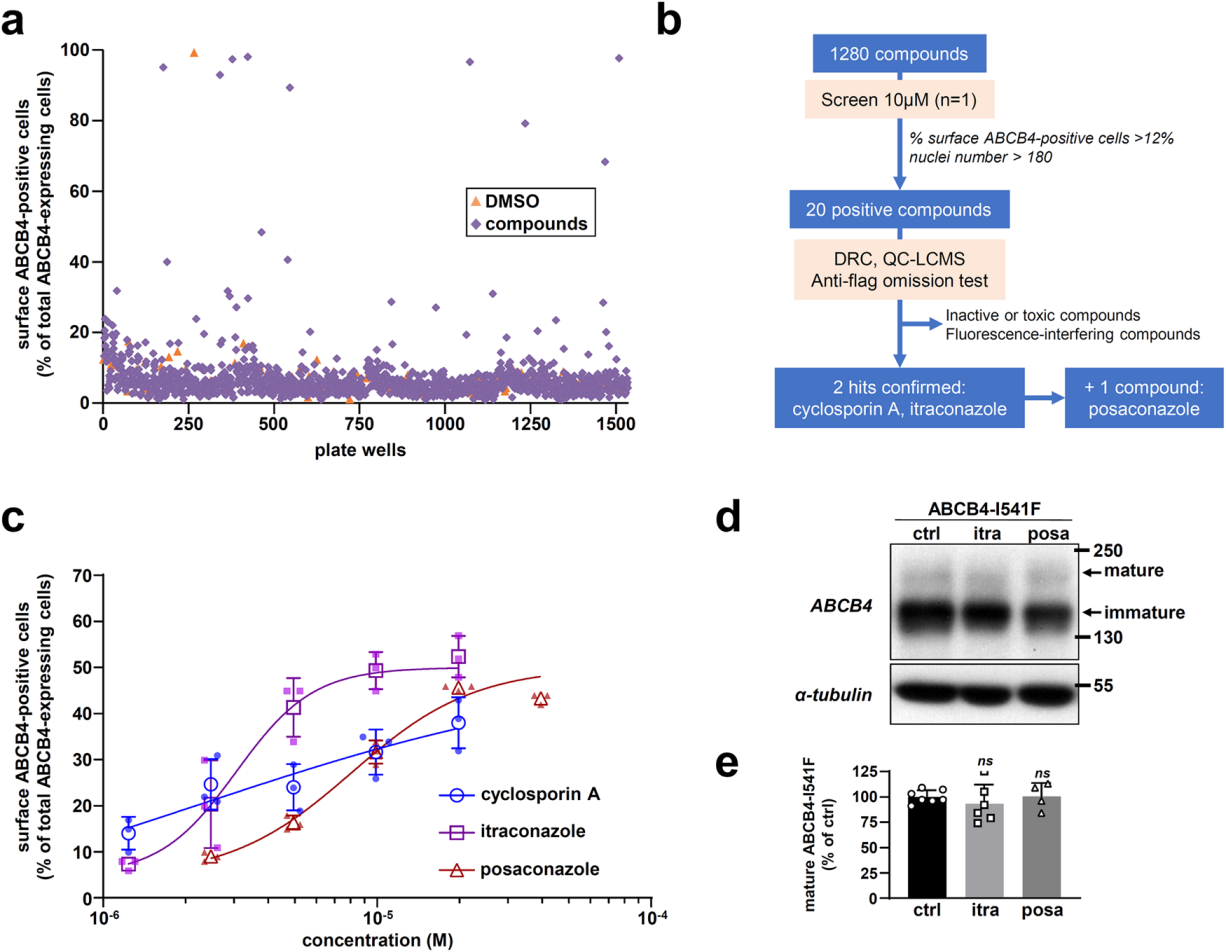
A first HCS with an FDA-approved chemical library. **a** Overview of the screening with the percentage of surface ABCB4-expressing cells reported for each molecule: HEK cells stably expressing mCherry-ABCB4-FLAG-I541F were incubated for 16 h with 1280 compounds from the Prestwick chemical library® at 10 µM. DMSO was used as a vehicle negative control. **b** Workflow of the HCS campaign. DRC: dose-response curve; QC-LCMS: quality control by liquid chromatography-mass spectrometry. **c** Dose-response curves of the three compounds of interest using the primary screening model. Percentages of cells with surface ABCB4 were determined and reported as means (± SD, *n* = 3). **d** HEK cells transiently expressing mCherry-ABCB4-FLAG-I541F were treated for 16 h with DMSO (ctrl), 10 µM itraconazole (itra) or 10 µM posaconazole (posa). ABCB4-I541F maturation was assessed by immunoblot as in Fig. 1b. Molecular weight markers (in kDa) are indicated. This panel is representative of four independent experiments. Full immunoblots are shown in Supplementary Fig. 14b. **e** Densitometry analysis of (**d**). The amount of ABCB4-I541F mature form was quantified, normalized to the amount of tubulin and then expressed as a percentage of vehicle-treated cells (ctrl). Means (± SD) of at least four independent experiments per condition are shown.

We first used our cellular model to screen the Prestwick chemical library^®^, which is a collection of 1280 FDA-approved chemical compounds with high chemical and pharmacological diversity. Drug repurposing to treat pathologies other than their initial indication has become an attractive idea since it might accelerate drug development steps and reduce risks of failure^26^. The compounds were screened at the concentration of 10 µM for their capacity to correct plasma membrane localization of mCherry-ABCB4-FLAG-I541F in HEK cells (Fig. 2a, Supplementary Table 1, Supplementary Fig. 3). Two thresholds were set to select primary positive hits: 1) more than 12% of surface ABCB4-positive cells was required; 2) the number of nuclei counted was at least identical to that of the CsA-treated condition (> 180), indicating that there was no major toxicity. Thus, 20 compounds were selected and advanced in confirmatory steps, including (1) dose-response effect measurement using library stock solutions and (2) confirmation of the identity and purity of library samples (Fig. 2b). In addition, we checked that these hits did not induce fluorescent bias by testing them without anti-FLAG antibody. After discarding inactive, toxic or fluorescence-interfering compounds, two hits were identified: CsA (31.6 ± 4.9% surface ABCB4-positive cells at 10 µM) and itraconazole (49.3 ± 4.0% surface ABCB4-positive cells at 10 µM) (Fig. 2c). Confirming CsA as a hit compound present in the Prestwick library, as already reported^22^, supports our experimental approach. To strengthen this analysis besides the frame of this chemical library, we investigated the effect of posaconazole, another antifungal triazole compound structurally close to itraconazole. At 10 µM, posaconazole gave 31.7 ± 2.5% surface ABCB4-positive cells in our HCS model (Fig. 2c). However, itraconazole and posaconazole were not able to correct ABCB4-I541F maturation (Fig. 2d; quantification in Fig. 2e). Given that these compounds were also reported as ABCB4-mediated PC secretion inhibitors^27–29^, they were not further investigated as drug candidates. These results confirmed the relevance of our HCS strategy for further screening.

### Identification of new correcting hits from an additional high-content screening

**Fig. 3:**
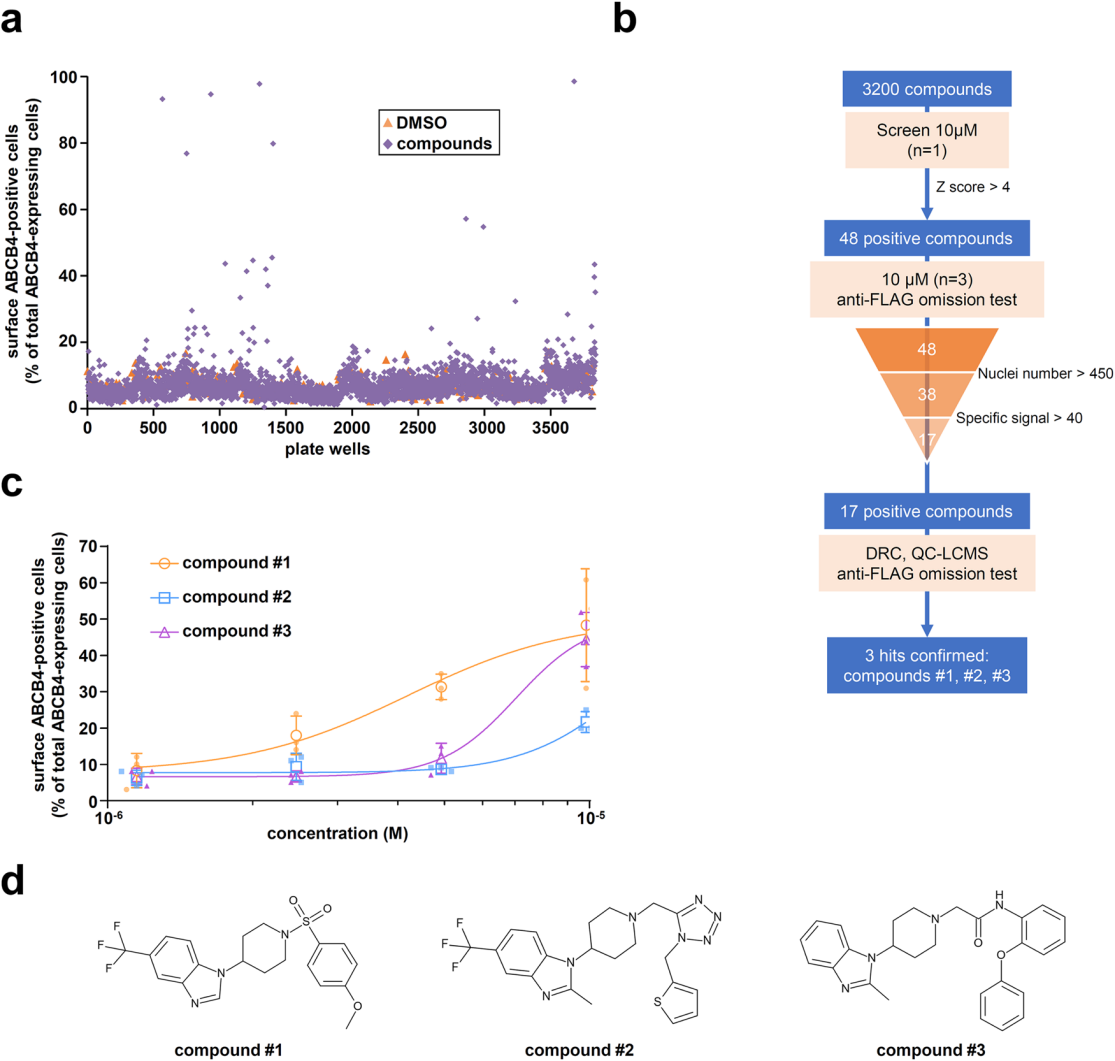
Identification of new ABCB4 correctors through additional HCS. **a** Overview of the screening in HEK cells stably expressing mCherry-ABCB4-FLAG-I541F, as in Fig. 2a: 3200 compounds were tested at 10 µM for 16 h. DMSO was used as a vehicle negative control. **b** Workflow of the HCS campaign, as in Fig. 2b. **c** Dose-response curves of the three hits, performed as in Fig. 2c. **d** Structure of ABCB4 correctors identified by HCS.

A home-made library of 3200 compounds, selected from commercial vendors or prepared by our chemists to maximize diversity and fulfill “drug/lead-likeness” structural requirements, was screened at 10 µM for their capacity to correct plasma membrane localization of mCherry-ABCB4-FLAG-I541F in HEK cells (Fig. 3a). As positive controls, we systematically kept CsA and itraconazole (see previous section). The strictly standardized mean differences (SSMD) were calculated for each plate and ranged from 3.06 to 4.72 (Supplementary Fig. 3), indicating a robust assay and authorizing us to proceed with data analyses for the compounds. We categorized the compounds based on their Z-score (Supplementary Fig. 4). The higher the Z-score, the higher the corrected mCherry-ABCB4-FLAG-I541F protein expressed at the cell membrane. Forty-eight compounds with a Z-score higher than 4 were selected and tested in triplicate at 10 µM in the HCS assay (Fig. 3b). In addition, the absence of hit-induced fluorescent bias was evaluated without anti-FLAG antibody. Finally, 17 positive compounds were then selected since they (i) were associated with highly specific anti-FLAG intensity signals (the difference of test signal with background signal was > 40) and (ii) did not exhibit cytotoxicity (nuclei number > 450) (Supplementary Fig. 5). The identity and purity of these compounds were confirmed by liquid chromatography-coupled mass spectrometry. These 17 compounds were tested again at varying concentrations, but only the activity of three molecules could be confirmed: compounds #1, #2 and #3 rescuing 48.3 ± 15.5%, 21.7 ± 2.9% and 44.3 ± 7.5% of surface ABCB4-positive cells at 10 µM, respectively (Fig. 3c, d). These apparent incomplete rescue efficiencies of plasma membrane targeting after hit treatment may be due to sub-optimal compound concentrations or fluorescence thresholds applied to an heterogenous cell population expressing ABCB4 at different levels. In the following sections, we describe the characterization of these three hits.

### Validation of the correcting hits on the traffic and localization of the ER-retained ABCB4-I541F variant

**Fig. 4:**
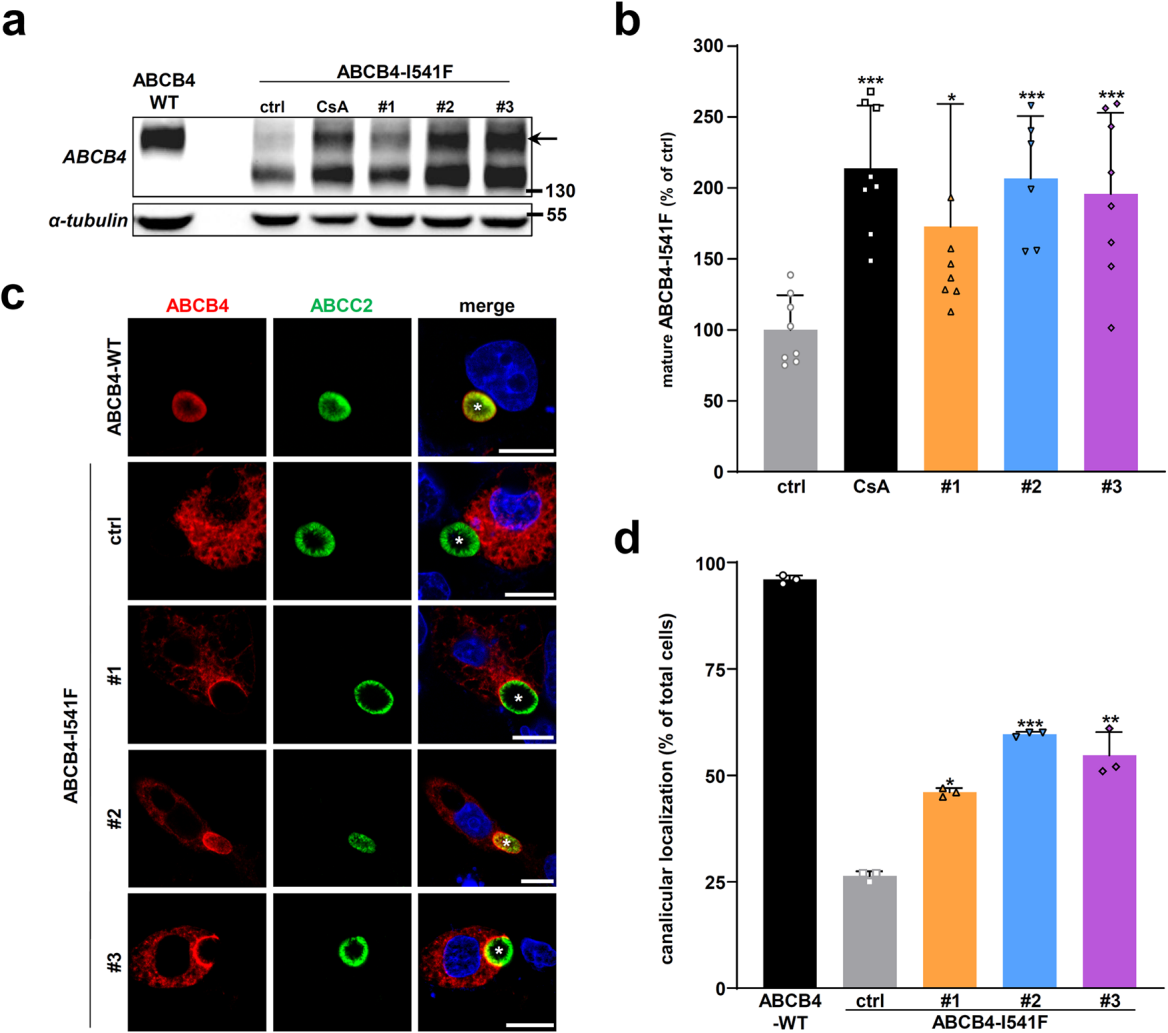
The HCS hits partially correct the maturation and canalicular targeting of ABCB4-I541F. **a** HEK cells transiently expressing ABCB4-I541F were treated for 16 h with vehicle (ctrl, DMSO) or 10 μM of the three indicated hits. ABCB4-I541F maturation was assessed by immunoblot as in Fig. 1b. The arrow indicates mature ABCB4. This panel is representative of at least six independent experiments for each condition. Full immunoblots are shown in Supplementary Fig. 14c. **b** Densitometry analysis of (**a**). The amount of ABCB4-I541F mature form was quantified, normalized to the amount of tubulin and then expressed as a percentage of vehicle-treated cells (ctrl). Means (± SD) of at least six independent experiments per condition are shown. **c** ABCB4-WT or ABCB4-I541F were transiently expressed in HepG2 cells. After 16 h of treatment with vehicle (ctrl, DMSO) or 10 µM of the indicated hits, cells were fixed and permeabilized. Cellular localization of ABCB4 (red) and the canalicular transporter ABCC2 (green) was then visualized by confocal microscopy after indirect immunofluorescence labeling. Nuclei shown in the merged images were labeled with Hoechst 33342 (blue). White asterisks indicate bile canaliculi. This panel is representative of three independent experiments. Bars: 10 µm. **d** Quantification of ABCB4-I541F canalicular localization from c. ABCB4 colocalization with ABCC2 was determined in 300 cells for each condition. Means (± SD) of three independent experiments are shown.

The potential cytotoxicity of the three selected hits was assessed using HEK cell viability assays for 16 h. No significant toxicity was reported for all candidate molecules up to 25 µM (Supplementary Fig. 6). Then, to validate the correcting effect of the three hits, in vitro analyses in non-polarized (HEK) and polarized (HepG2) cell models were conducted using the prototypical class II ABCB4-I541F variant. Immunoblots on HEK cell lysates expressing ABCB4-I541F showed that the compounds #1, #2 and #3 at 10 µM partially, but significantly, corrected protein maturation, as efficiently as 10 µM CsA (Fig. 4a; quantification in Fig. 4b).

The subcellular localization of ABCB4-I541F was investigated in polarized HepG2 cells after treatment with the hits at 10 µM. ABCB4-WT exhibited a nice colocalization (> 95%) with the canalicular marker ABCC2, while ABCB4-I541F remained mostly cytoplasmic, indicating its intracellular retention (Fig. 4c; quantification in Fig. 4d). Treatment with compounds #1, #2 and #3 partially restored the canalicular targeting of ABCB4-I541F (Fig. 4c; quantification in Fig. 4d), in agreement with the previous immunoblot analyses (Fig. 4a, b).

### Effect of the correcting hits on the secretory function of ABCB4-I541F and ABCB4-WT

**Fig. 5:**
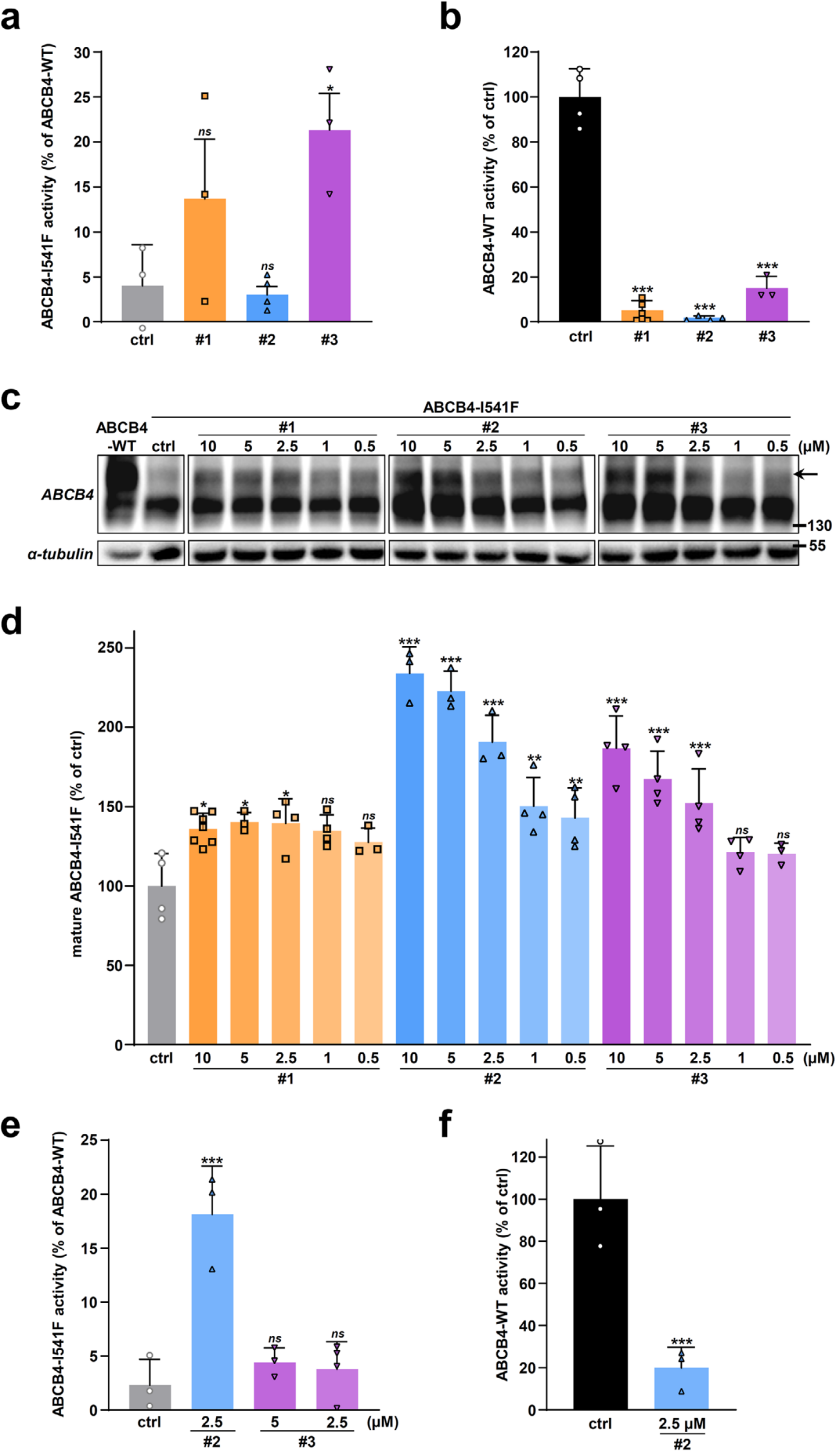
Effect of the hits on the function of ABCB4-I541F and ABCB4-WT. **a**, **b** HEK cells expressing ABCB4-I541F (**a**) or ABCB4-WT (**b**) were treated with vehicle (ctrl, DMSO) or 10 µM of the indicated hits. ABCB4-mediated PC secretion was measured, normalized to ABCB4 expression levels, and expressed as a percentage of the activity of vehicle-treated cells expressing ABCB4-WT. Means (± SD) of at least three independent experiments performed in triplicate for each tested condition are shown. **c** After transient expression of ABCB4-I541F, HEK cells were treated with decreasing doses of the indicated drugs for 16 h. Then, immunoblot analyses were performed to assess ABCB4-I541F maturation as in Fig. 4a. This panel is representative of three independent experiments. Full immunoblots are shown in Supplementary Fig. 14d. **d** Densitometry analysis of (**c**). The percentage of ABCB4-I541F mature form was determined and represented as in Fig. 4b. Means (± SD) of at least three independent experiments per condition are shown. **e**, **f** ABCB4-mediated PC secretion was determined as in (**a**, **b**) for ABCB4-I541F (**e**) and ABCB4-WT (**f**) -expressing cells treated with 2.5 or 5 µM of the indicated hits. Means (± SD) of at least three independent experiments performed in triplicate for each tested condition are shown.

In line with our previous studies^19^, the function of class II ABCB4 variants investigated until now was at least partially restored once addressed at the plasma membrane. Therefore, our strategy aims to correct a sufficient PC secretion into the extracellular medium in cell models. PC secretion after treatment was thus monitored. Using 10 µM for the three hits, a partial but significant restoration of PC secretion activity of ABCB4-I541F was observed with compound #3 (21.3 ± 7.0% of ABCB4-WT activity), while only a non-significant tendency to restore was observed with compound #1 and no restoration with compound #2, 13.7 ± 11.5% and 3.0 ± 1.8% of ABCB4-WT activity, respectively (Fig. 5a). The last observation is in striking contrast with the observed efficiency of compound #2 in partially rescuing ABCB4-I541F maturation and canalicular localization (Fig. 4). We then analyzed ABCB4-WT-mediated PC secretion after treatment with the three hits and we observed an important inhibitory effect, which was stronger with compound #2 and milder with compound #3 (Fig. 5b), 1.8 ± 0.9% and 15.0 ± 5.2% of ABCB4-WT activity, respectively, in agreement with the restoration efficacy of these three drugs on ABCB4-I541F function (Fig. 5a).

In order to circumvent this inhibition effect on ABCB4 function, further investigations were carried out using lower drug concentrations to obtain better correction/inhibition ratios. Dose-response analyses were performed using 0.5 to 10 µM for each molecule and ABCB4-I541F maturation was assessed by immunoblot: an important dose-dependent ABCB4-I541F maturation correction (but partial compared with ABCB4-WT mature form) was observed with compounds #2 and #3 (Fig. 5c). Quantification of these experiments (Fig. 5d) showed concentration dependency leading to the selection of compound #2 at a concentration of 2.5 µM and compound #3 at a concentration of 5.0 and 2.5 µM for further analyses. Nevertheless, in functional assays with compound #3, the restoration of PC secretion activity of ABCB4-I541F was lost at 5.0 and 2.5 µM (Fig. 5e, 4.4 ± 1.4% and 3.8 ± 2.6% of ABCB4-WT activity, respectively) as compared with a 10 µM treatment (Fig. 5a). Interestingly, after treatment with 2.5 µM of compound #2, a partial but significant restoration of ABCB4-I541F function was observed (Fig. 5e, 18.1 ± 4.5% of ABCB4-WT activity), in contrast to what we observed at 10 µM (Fig. 5a, 3.0 ± 1.8% of ABCB4-WT activity). We then tested the effect of compound #2 at 2.5 µM on ABCB4-WT activity and lower ABCB4-WT function inhibition was observed as compared to 10 µM (Fig. 5f, 19.9 ± 9.8% of remaining ABCB4-WT activity; compare with Fig. 5b).

These results highlight the potency of the identified hits to correct ABCB4 maturation and traffic that are counterbalanced by their inhibitory effect on the function of the transporter. However, for compound #2, the latter can be reduced by lowering drug concentration.

### Analysis of the correction efficacy of the hits on two other ER-retained ABCB4 variants

**Fig. 6:**
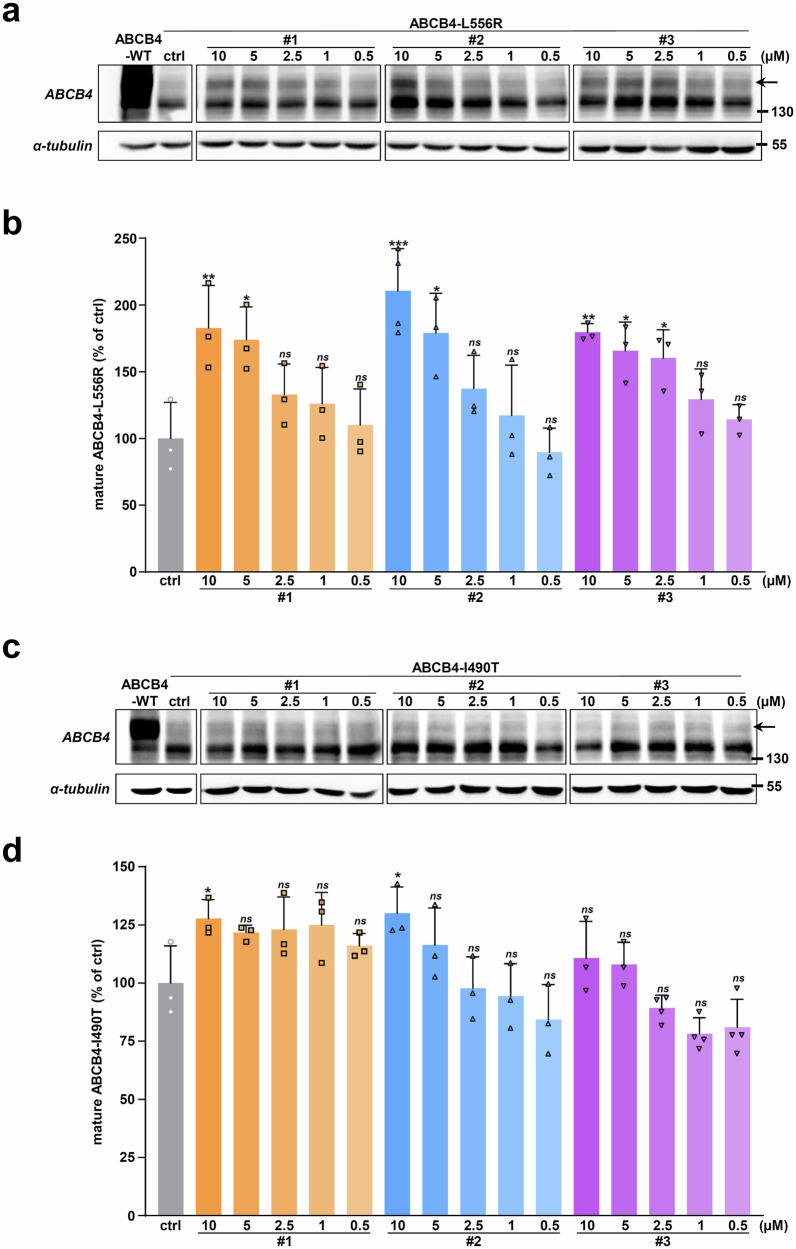
Effect of the HCS hits on the maturation of two other ER-retained ABCB4 variants. **a** ABCB4-L556R-expressing HEK cells were treated with vehicle (ctrl, DMSO) or decreasing doses of the indicated hits for 16 h. Then, immunoblot analyses were performed to assess ABCB4 maturation as in Fig. 4a. **b** Densitometry analysis of (**a**). The percentage of ABCB4-L556R mature form was determined and represented as in Fig. 4b. **c**, **d** The same analyses as in (**a**, **b**) were performed on ABCB4-I490T-expressing HEK cells. **a**, **c** are each representative of three independent experiments. For (**a**, **c**), full immunoblots are shown in Supplementary Fig. 14e, f. (**b,** **d**) represent means (± SD) of at least three independent experiments per condition.

**Fig. 7:**
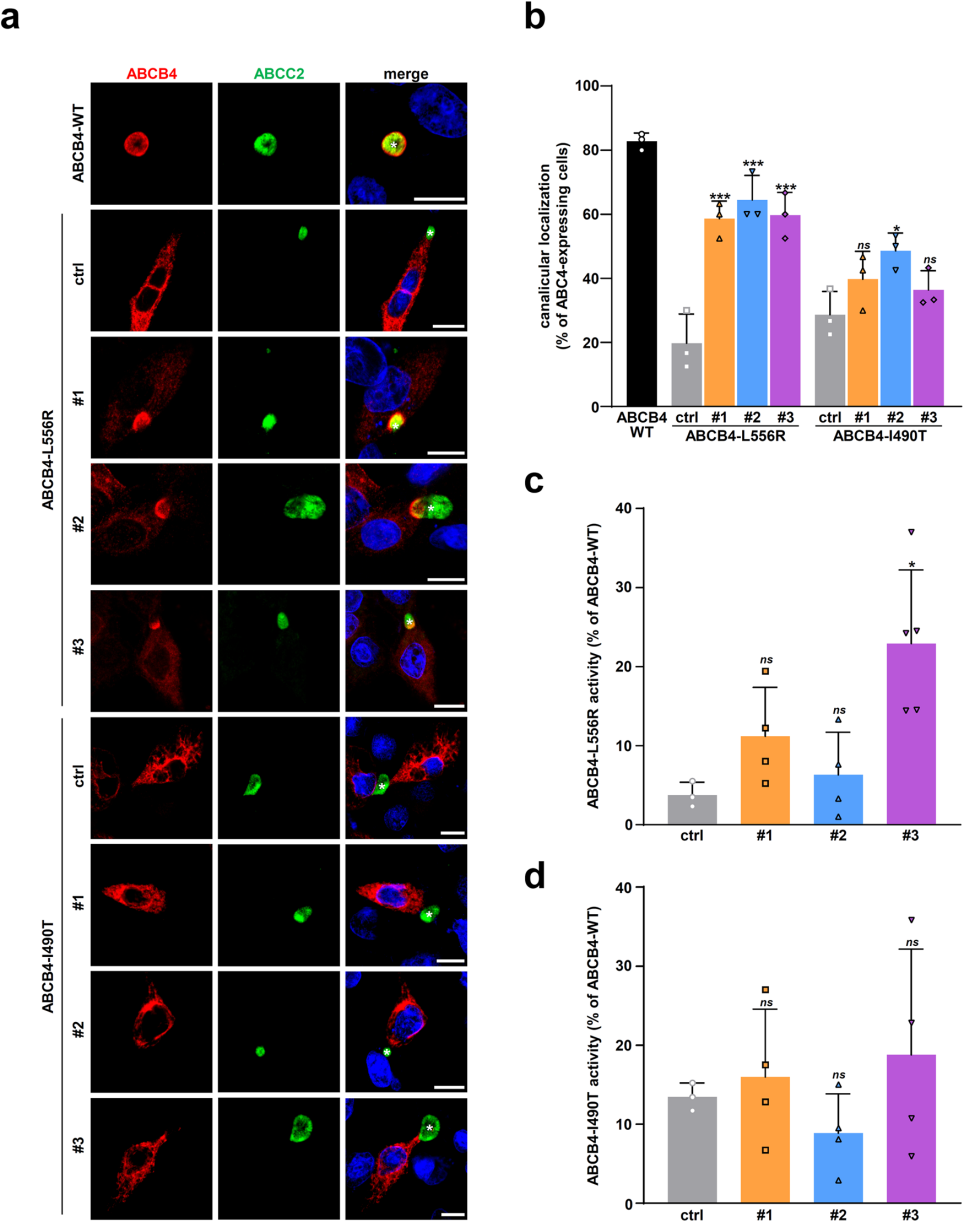
Effect of the hits on the traffic and function of ABCB4-L556R and ABCB4-I490T variants. **a** The subcellular localization of ABCB4-L556R and ABCB4-I490T was analyzed in HepG2 cells as in Fig. 4c, after treatment with vehicle (ctrl, DMSO) or 10 μM of the indicated hits. White asterisks indicate bile canaliculi. This panel is representative of three independent experiments. Bars: 10 μm. **b** The canalicular localization of ABCB4-L556R and ABCB4-I490T was determined from a, as in Fig. 4d. Means ( ± SD) of three independent experiments are shown. **c**, **d** The PC secretion activity of ABCB4-L556R (**c**) and ABCB4-I490T (**d**) expressed in HEK cells was determined as in Fig. 5a, b, after treatment with 10 µM of the indicated hits. Means ( ± SD) of at least three independent experiments performed in triplicate for each tested condition are shown.

After evaluating compounds #1, #2 and #3 on the ABCB4-I541F variant, we extrapolated our studies towards the correction efficacy analysis of these compounds on two other class II ER-retained ABCB4 variants, namely L556R and I490T^16,18,19,30^, described in patients with liver diseases^12,31^. We performed dose-response immunoblot analyses of the maturation correction for these two variants after treatment with the three hits. Our results indicate a significant dose-dependent correction of ABCB4-L556R maturation (Fig. 6a; quantification in Fig. 6b), but partial compared with ABCB4-WT mature form. But for ABCB4-I490T, only a modest tendency of maturation correction upon treatment with the three hits was observed (Fig. 6c; quantification in Fig. 6d). As performed before for ABCB4-I541F (Fig. 4c, d), subcellular localization analyses of these two variants in HepG2 cells showed that the three hits were able to partially correct ABCB4-L556R targeting to bile canaliculi (Fig. 7a; quantification in Fig. 7b). However, the canalicular localization correction was lower for ABCB4-I490T variant (Fig. 7a; quantification in Fig. 7b), in agreement with immunoblot analyses (Fig. 6c, d).

Finally, the secretory PC function of both ER-retained ABCB4 variants was assessed after drug treatment. The effect of compounds #1, #2 and #3 was tested at a single concentration of 10 µM in functional assays, given the robustness of the effect at this concentration observed on immunoblots compared to lower doses (Fig. 6). As observed with ABCB4-I541F (Fig. 5a), only compound #3 was able to partially but significantly restore ABCB4-L556R function (Fig. 7c, 22.9 ± 9.3% of remaining ABCB4-WT activity). Moreover, consistently with the results obtained in maturation and localization assays (Figs. 6c, d and 7a, b), ABCB4-I490T function was not corrected by the three compounds (Fig. 7d, 16.0 ± 8.5%, 8.9 ± 5.0%, 18.8 ± 13.4% of remaining ABCB4-WT activity for compounds #1, #2 and #3, respectively). These results indicate that only compound #3 at 10 µM is able to partially restore the maturation, localization and function of the ER-retained ABCB4-L556R variant, while no compound is able to significantly correct the other ABCB4-I490T variant.

### Potential interaction of the hits with ABCB4-WT revealed by molecular docking calculations

**Fig. 8:**
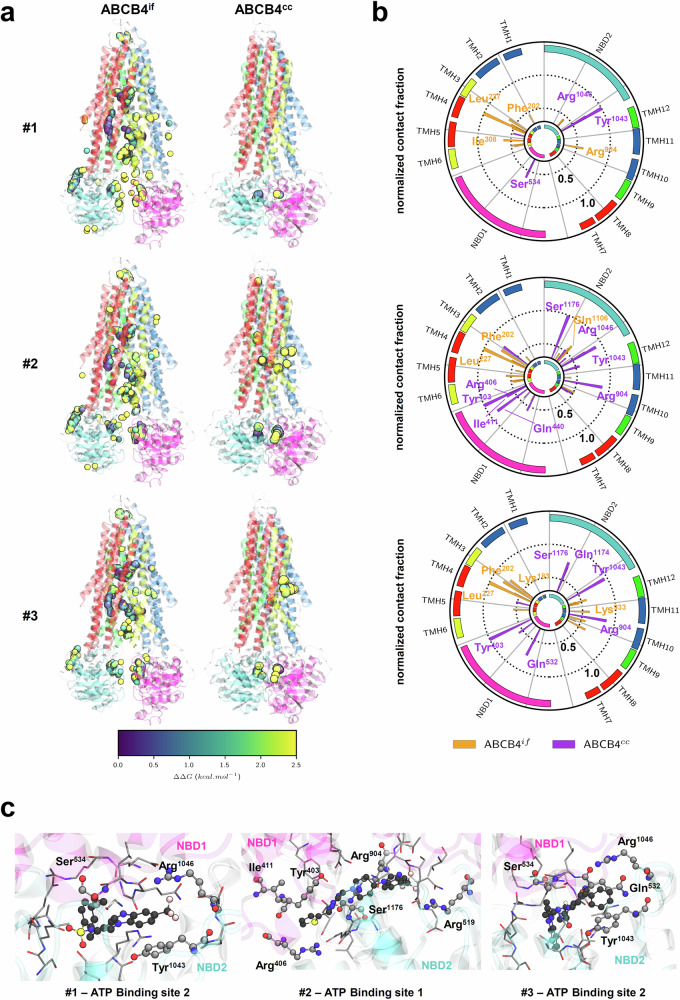
Potential interaction of the correcting hits with ABCB4 revealed by molecular docking calculations. **a** The selected centers of mass for compounds #1, #2 and #3 were obtained from brute force molecular docking calculations for which cutoff of 2.5 kcal.mol^-1^ was applied. Centers of mass were colored considering the lowest score affinity at 0.0 kcal.mol^-1^. **b** The most frequent contact residues were obtained by considering all selected poses displayed in (**a**). Given the large variability between selected number of poses, contact fractions were normalized as 1.0 for the most frequently observed residue per conformation. ABCB4^if^ and ABCB4^cc^ residues were depicted in orange and purple, respectively. **c** Examples of the lowest affinity scores for compounds #1 (left), #2 (center) and #3 (right) in the ATP-binding sites. Small molecules and key residues reported in b are shown in balls-and-sticks. Other residues with a contact distance lower than 4 Å are shown in licorice representation. For all panels, ABCB4 color code refers to as TMHs and NBDs.

Owing to the absence of cryo-EM resolved structure of ABCB4 variants, we here investigated possible direct interactions between compounds #1, #2, #3 and ABCB4-WT by means of in silico brute force molecular docking calculations. Binding modes with either inward-facing or close-cleft ABCB4-WT conformations (ABCB4^if^ and ABCB4^cc^, respectively) were considered. A total of 266,253 molecular poses were initially obtained for all ligands and the two ABCB4 conformations (Supplementary Table 2). Binding affinity scores range from -12.2 to +2.4 kcal.mol^-1^ (Supplementary Fig. 7 and Supplementary Table 2). A binding affinity score cutoff of 2.5 kcal.mol^-1^ was applied, reducing to 6455, 5156, and 3098 selected poses in ABCB4^if^ for compounds #1, #2, and #3, respectively, while 325, 1304, and 829 selected poses were obtained with ABCB4^cc^ for compounds #1, #2, and #3, respectively. Regardless of ABCB4 conformation, the three compounds exhibit similar binding regions (Fig. 8a; detailed regions in Supplementary Table 3). More binding regions were systematically predicted for ABCB4^if^ than ABCB4^cc^. This might be explained by the wider space owing to larger ABC intracellular angle (Fig. 8a)^32,33^. Globally, affinity scores are more favorable for binding to ABCB4^cc^ than ABCB4^if^, selected best poses affinity ranging from −12.2 to −10.9 and from −10.2 to −9.4 kcal.mol^−1^, respectively, for the three molecules (Supplementary Fig. 7 and Supplementary Table 2). For ABCB4^if^ and ABCB4^cc^ conformations, molecular docking calculations suggest that binding of compound #3 (best score = −12.2 kcal.mol^−1^) is slightly more favorable than compounds #1 and #2, the last two exhibiting similar affinity scores (best scores = −10.9 and −11.0 kcal.mol^−1^, respectively). At this stage, it is important to note that such molecular docking calculations were performed with ABCB4-WT and should be carefully considered for variants which would be associated with severe structure unfolding.

In ABCB4^if^, molecular poses sample over the (i) intracellular protein chamber as well as the so-called front gate for PC channel access between transmembrane helix (TMH) 4 and TMH6^34^, (ii) ATP-binding sites, (iii) interface between extracellular loops of TMH1-2 and TMH3-6 and (iv) intracellular loop connecting TMH12 and nucleotide-binding domain (NBD) 2 (Fig. 8a). Calculated affinity scores suggest that molecular poses in ATP-binding site 2, protein chamber, and PC channel access are slightly more likely. Contact analyses suggested residues which may be involved in hit binding. For instance, molecular docking calculations suggested Lys183, Phe202, Leu227, Ile308, Arg904, Lys933 and Gln1106 to participate in the binding of the three hits (Fig. 8b; see Supplementary Table 4 for details).

In ABCB4^cc^, most of the binding poses were observed in ATP-binding sites. Few molecular poses were observed in the protein chamber but associated with less favorable affinity scores (yellowish centers of mass in Fig. 8a). All three compounds tend to preferentially bind ATP-binding site 2. Contact-wise, both ATP-binding site A-loop aromatic residues were suggested to be involved in hit molecule binding by means of π-stacking interactions, namely Tyr403 and Tyr1043 for ATP-binding sites 1 and 2, respectively (Fig. 8b; details in Supplementary Table 5). ABC-signature Ser1176 is also involved in hit binding as well as cationic arginines, e.g. Arg406, Arg904 and Arg1046 (Fig. 8b; Supplementary Table 5). Compounds #1 and #2 exhibit similar binding patterns in which π-stacking interactions were obtained between ligand purine-like moiety and A-loop aromatic residues (Fig. 8c). Compounds #1 and #2 binding modes are also strengthened thanks to the electronegative trifluoromethyl moiety (see Fig. 3d) with surrounding cationic arginine residues, e.g. Arg1046 or Arg529/Arg904 for ATP binding sites 2 and 1, respectively (Fig. 8c). In the absence of trifluoromethyl moiety, compound #3 adopts another preferential binding mode in which oxydibenzenyl moiety (see Fig. 3d) interacts with the A-loop aromatic residue Tyr1043 through π-stacking interactions (Fig. 8c).

For the sake of comparison, potential binding sites were also predicted individually for each protein using the PUResNET and PrankWEB online tools^35,36^. PUResNET predicted only two binding sites for each conformation (Supplementary Fig. 8), whereas PrankWEB suggested up to eighteen potential binding sites (Supplementary Fig. 9 and Supplementary Tables 6 and 7). However, for our study, only the first ten were considered, corresponding to a probability higher than 0.1. It is important to note that, like molecular docking calculations, these results must be approached with caution. For ABCB4^if^, molecular docking poses were observed in the predicted binding sites obtained from both PUResNET and PrankWEB predictions (Supplementary Figs. 10 and 11). Importantly, a significantly higher agreement was observed with the latter, as PUResNET only predicted two binding sites located in TMDs. Similarly, molecular poses obtained for the ABCB4^cc^ conformation showed good agreement with protein-based binding site predictions. ATP-binding sites were predicted by both approaches to be the most likely binding sites for compounds #1, #2, and #3. Importantly, molecular docking poses which were not in line with PUResNET and PrankWEB predictions are located at the protein interface with its environment.

## Discussion

Genetic variations of ABCB4, the hepatobiliary transporter of phospholipids, are associated with several rare cholestatic diseases, the most severe form being PFIC3^10^. These genetic variations can affect the transporter at different levels: expression, intracellular traffic, function or stability^16,17^. Focusing on class II ABCB4 variants, which present trafficking defects with intracellular retention, several molecules have already been proposed as potential correctors and include structural analogues of roscovitine^19^, ABCC7/CFTR correctors^18^ and other small molecules (for a review, see ref. ^17^). Here, we described an original fluorescence-based assay that allowed us to screen thousands of compounds through a HCS approach. Using this technique, three hits were identified and confirmed as correctors of the maturation and the canalicular localization of two distinct class II ABCB4 variants, ABCB4-I541F and -L556R. It is interesting to note that the three hits were not able to significantly correct (or poorly corrected) the maturation and the canalicular localization of a third ABCB4 variant (ABCB4-I490T), suggesting that this mutation induces different protein folding defects, a different conformational landscape and weaker interactions with the molecules than in the case of I541F and L556R variants.

Regarding the PC secretory function of the transporter, we observed that only compound #3 at 10 µM was able to significantly increase PC secretion for ABCB4-I541F and -L556R variants, while this was a non-significant tendency with compound #1 at 10 µM. As expected from maturation and localization analyses, no significant functional restoration was observed for ABCB4-I490T with the three molecules. Strikingly, these three hits inhibited ABCB4-WT function at different levels, an effect that was minimized when hit concentration was lowered, which may explain why we observed an increase of ABCB4-I541F secretory function with compound #2 at 2.5 µM but not at 10 µM. We have previously reported such partial or total inhibition of ABCB4-WT function with roscovitine analogues or ABCC7/CFTR correctors, respectively^18,19^, an effect that was previously reported for CsA, which was identified as an ABCB4 corrector^22^ but further shown to be an inhibitor of the transporter’s function^28^. Here, we observed that compound #3 was not able to rescue ABCB4-I541F function when used at 5 µM while this concentration allowed a maturation rescue close to the one observed after treatment at 10 µM. The reason for such discrepancy is unclear but we may speculate that it could be due to the fact that these sets of experiments are independent and thus subject to fluctuations in rescue efficiency. This could also be explained by the uncoupling of the traffic and function of the transporter, as we previously proposed^18,19^: a dose allowing trafficking/maturation rescue may not be sufficient to allow a partial but significant restoration of function. It is thus tempting to speculate that on the one hand all these correctors help, directly or indirectly, a better folding of ABCB4 defective variants which can then escape ER-associated degradation and be further processed to the plasma membrane. On the other hand, the properly folded variants would not be functional enough to secrete normal PC levels after reaching the plasma membrane. As suggested by molecular docking calculations, it may be explained by the direct interaction of the small molecules with the transporter. Assuming here molecular docking calculations as a predictive approach, our calculations indicate that the three hits may interact with A-loop aromatic residues (Tyr403 and Tyr1043) required for ATP binding in ABC transporters, including ABCB4^37^, which may in turn preclude ATP binding and/or hydrolysis required for proper ABCB4 function. This may be paralleled with the observed inhibitory effect on ABCB4-WT. For instance, compound #3 exhibits lower ABCB4-WT functional inhibition and the best molecular pose suggests an inverted binding mode than compounds #1 and #2 in ABCB4^cc^ conformation. Even if such investigation would require substantial amounts of work and expertise, predictive studies can be considered as a first step, employing in silico molecular docking analyses as a useful tool. These calculations may be helpful to guide structure-activity analyses and chemical optimization in order to reach better correction/inhibition ratios by lowering the ATP-binding site affinity in the context of medicinal chemistry strategies. We would like to emphasize that these molecular docking calculations are only predictions of the potential interactions of the compounds with ABCB4-WT but not with the variants, since their 3D structures are not resolved yet. Indeed, it is possible that the hits identified here may interact with different regions of the variants, due to conformational changes induced by the mutations, which may explain their inhibitory effect on ABCB4-WT *vs* their stimulatory effects on two variants at certain concentrations. But if the compounds interact in a similar manner with ABCB4-WT and the variants (which is not proven here – see above), it may highlight that the residual function observed for ABCB4-WT after drug treatment is enough to observe a partial restoration of function for the variants. Because the three hits may bind to ATP-binding sites, an interesting perspective would be to investigate the effect of non-hydrolysable ATP analogues on the intracellular traffic and maturation of class II ABCB4 variants. It is worth mentioning that correctors and potentiators clinically used for e.g., CFTR-F508del mutation were recently shown by means of cryo-EM resolution to bind to unexpected regions distant from mutation site, TMD central cavity or ATP-binding sites^38^, in contrast to our present results. These findings shed light on the complexity of small molecule interactions with ABC protein mutants. If we hypothesize that ABCB4-I541F and L556R are not associated with an important misfolding, the presence of benzimidazole moiety in compounds #1, #2 and #3 may favor interactions with ATP-binding site A-loop motif in contrast to CFTR modulators. However, it is important to note that molecular docking poses were located at the interface between ABCB4 and high density polar head lipid region as observed for cryo-EM-resolved CFTR bound to elexacaftor, tezacaftor and ivacaftor^38^. This may pave the way for further drug design by: *i)* modifying benzimidazole moiety to decrease ring planarity, and *ii)* improving interaction with lipid polar head region. It is thus important to also consider alternative binding sites, such as those located at the ABCB4-lipid interface, as observed for the interaction of elexacaftor, tezacaftor, and ivacaftor with CFTR^38^.

Concerning the therapeutic interest of the newly identified molecules, compounds #1, #2 and #3 constitute interesting candidates since they allowed a partial correction of ABCB4-I541F and L556R maturation, canalicular localization and/or function. Even if the correction is partial, it may be sufficient to make patients responsive to already existing treatments. Indeed, it has recently been shown that PFIC3 patients are better responders to ursodeoxycholic acid treatment when phospholipids represent at least ~7% of total biliary lipids^39^. Thus, even if the restoration of function of ABCB4 variants is only partial after treatment with correctors, this may be sufficient to rescue PC secretion into bile above this threshold and thus make patients responders to other therapeutic molecules such as ursodeoxycholic acid. In the same manner, the newly identified compounds may be combined with potentiators, as performed for cystic fibrosis patients carrying the F508del mutation^40,41^, since the potentiator VX-770/ivacaftor has been shown to partially correct the function of activity-defective class III ABCB4 variants^42,43^. However, the rescuing efficacy of the newly identified ABCB4 correctors will have to be confirmed on other class II ABCB4 variants in vitro and further validated in vivo in preclinical mouse models before considering their transfer to the clinic. Ultimately, the use of these new correctors may be broadened to traffic-defective variants of other ABC transporters, including ABCB11/bile salt export pump^44^.

To conclude, we provide here a new HCS tool that helped us to identify new potential correctors for rare ABCB4-related cholestatic diseases such as PFIC3. While these molecules would be of therapeutic interest, future work will be necessary in order to chemically optimize these drug candidates before considering their clinical use.

## Methods

***Additional materials and methods are described in Supplementary Materials and Methods***.

***Datasets supporting graphs are available in*** Supplementary Data 1*.*

### Plasmids, cell culture, transfection and cell treatments

The pcDNA3 plasmid encoding ABCB4-WT has been described in ref. ^21^. The I541F, I490T, and L556R missense ABCB4 variants and encoding plasmids were also reported and described in refs. ^12,16,19,21,30,31^. Human embryonic kidney (HEK-293, herein referred to as HEK; ATCC®-CRL-1573^TM^) cells and human hepatocellular carcinoma HepG2 (ATCC®- HB-8065^TM^) cells were obtained from ATCC (Manassas, VA, USA) and grown at 37 °C under a humid atmosphere with 5% CO_2_, as previously described in refs. ^18,19^. Transient transfections with plasmids (1 or 2 µg DNA for a well of a 6 well-plate for HEK cells and HepG2 cells, respectively) were performed 6 (HEK cells) or 24 (HepG2 cells) hours after seeding using FuGENE^®^ HD (Promega France, Charbonnières-les-Bains, France) for HEK cells and JetPrime (PolyPlus Transfection, Illkirch, France) for HepG2 cells, following manufacturers’ instructions.

For HCS, ABCB4-FLAG-WT with a FLAG tag (DYKDDDDK) within its first extracellular loop between Ser 99 and Leu 100 (already described in ref. ^18^), was subcloned into pmCherry-C1 (Takara Bio Europe, Saint-Germain-en-Laye, France) using the unique HindIII and XbaI restriction sites (Genscript, Piscataway, NJ, USA). Then I541F targeted mutagenesis was performed as published^21^. The sequences of the final constructs, pmCherry-ABCB4-FLAG-WT and pmCherry-ABCB4-FLAG-I541F (see Fig. 1a), were verified by automated Sanger sequencing. These constructs were stably expressed in HEK cells after puromycin selection (1 µg/ml) and maintenance (0.3 µg/ml). Then, frozen stocks of total stable cell populations were constituted in order to perform the different HCS experiments with cells at the same passage.

### High-Content Screening and data processing

We screened the drug-repository Prestwick chemical library^®^ (Illkirch, France) and a library of 3200 compounds, selected from commercial vendors or prepared by our chemists using state-of-the-art selection and design criteria, in terms of diversity and “drug/lead-likeness” properties. Compounds in both libraries were stored at 10 mM in DMSO in 384-well Echo qualified source plates at −20 °C. For further characterization of hits, itraconazole and posaconazole were purchased from Clinisciences (Nanterre, France) and compounds #1, #2 and #3 from Asinex (Amsterdam, The Netherlands). Following discontinuation of compound #2 by commercial sources, it was resynthesized in-house (see Chemical synthesis in Supplementary Materials and Methods). CsA was from Euromedex (Souffelweyersheim, France).

At the beginning of the screening campaign, we dispensed 150 nL of compounds per well into 384-well plates using the ECHO550 nanoliquid handler (Beckman Coulter, Labcyte, Sunnyvale, CA, USA). These intermediate compound plates were frozen at −20 °C until use. mCherry-FLAG-ABCB4-I541F-expressing HEK cells were seeded in black 384-well, optically clear bottom, poly-L-lysine coated microplates at a density of 4000 cells per well in 40 µL medium. The next day, the intermediate compound plates were thawed and 30 µL medium was added in each well. Finally, 10 µL of the diluted compounds were distributed in the wells to achieve the target test concentrations in a final volume of 50 µL. Final concentration of DMSO did not exceed 0.1%. CsA alone or CsA and itraconazole were used as positive references in each plate during the first and second screening campaigns, respectively. Control mCherry-FLAG-ABCB4-WT-expressing HEK cells were seeded similarly in a separate plate and incubated with the vehicle. Incubates performed without anti-FLAG antibody indicated background signal (omission test). Assay microplates were then incubated for 16 h at 37 °C (5% CO_2_). Indirect immunofluorescence analyses were then performed as described in Supplementary Materials and Methods. Three images per well were acquired using the IN Cell Analyzer 6000 microscope (GE Healthcare Life Sciences, Buc, France) at 20X magnification in a confocal mode for Hoechst 33342 (ex.405/em.455 nm), or non-confocal mode for mCherry (ex.561/em.605 nm) and AlexaFluor^TM^488 (ex.488/em.525 nm), respectively. Images were analyzed with Columbus^TM^ software version 2.9.1 (Perkin Elmer Informatics, Waltham, MA, USA) using a customized script. In brief, after nuclei detection with Hoechst blue channel, cytoplasm was segmented and ABCB4-expressing cells were selected using the mCherry channel. Then, the subpopulation with surface ABCB4 was detected using the AlexaFluor^TM^488 (anti-FLAG) labeling and parameters of interest were quantified, including the percentage of surface ABCB4-positive cells and the nuclei number to evaluate cytotoxicity.

### In silico molecular docking and calculations

Two conformations of human ABCB4 were considered for molecular docking calculations, namely inward-facing and closed-cleft conformations (ABCB4^if^ and ABCB4^cc^, respectively). Both models were built from the resolved cryo-EM structures (PDB IDs: 7NIU^45^ and 6S7P^37^ for ABCB4^if^ and ABCB4^cc^, respectively) using a slightly different approach as that proposed previously^18^. To assess the binding site by molecular docking calculations, ATP molecules resolved in ABCB4^cc^ were not included. Further technical details about the preparation of ABCB4 models and ligands are reported in Supplementary Materials and Methods. Molecular docking calculations were carried out using the Vina-GPU 2.0 software^46,47^. Since no a priori knowledge was available regarding the binding modes of the compounds and ABCB4, a brute force approach was used. The overall search volumes comprising the whole ABCB4 protein structures were divided into 113 smaller sub-volumes (29 × 29 x 29 Å^3^, see Supplementary Fig. 12). For each grid and compound, 20 replicas of molecular docking calculations were performed. The maximum number of molecular poses was set to 20 by replica with an energy cutoff defined at 4 kcal.mol^−1^. The number of threads for molecular docking calculations was set up at 8000 to ensure sufficient computational effort for molecular pose search. Given the large number of possible flexible residues, we here only assume dihedral angle flexibilities for ligands (Supplementary Fig. 13). ABCB4 binding sites were also predicted using PUResNET (https://nsclbio.jbnu.ac.kr/tools/jmol - see Supplementary Figs. 8 and 10) and PrankWEB (https://prankweb.cz/ - see Supplementary Figs. 9 and 11) online tools^35,36^, using resolved cryo-EM ABCB4 structures. Regarding the latter, only binding pockets with a probability higher than 0.1 were considered for comparison with molecular docking calculations (see Supplementary Tables 6 and 7).

### Statistics and Reproducibility

Compounds were ranked according to the percentage of surface ABCB4-positive cells or based on their robust Z-score. This score was calculated using AlexaFluor^TM^488 (anti-FLAG) signal intensities and related to the median anti-FLAG intensity on their respective screening plate, according to the following equation, where x_i_ is the measured value, $\widetilde{{{{\rm{x}}}}}$ the median of the data set and MAD the median absolute deviation for the standard deviation:

$${robust\; Z}-{score}=\frac{{x}_{i}-\widetilde{x}}{{MAD}}$$

Graphics and statistical analyses (one-way ANOVA tests or Student’s *t*-test for Fig. 5f only) were performed using Prism version 7.00 (GraphPad software). Data are expressed as means ± standard deviation (SD). A *P*-value < 0.05 was considered significant with *P < 0.05; **P < 0.01; ***P < 0.005; ns: not significant. Symbols always indicate the comparison between the control (WT or vehicle-treated) and the other tested conditions. The number of experiments and/or replicates are indicated in figure legends.

### Reporting summary

Further information on research design is available in the Nature Portfolio Reporting Summary linked to this article.

### Supplementary information

Supplementary Information

Description of Additional Supplementary Materials

Supplementary Data 1

Reporting Summary

## Data Availability

The datasets generated and analyzed during the current study are available from the corresponding author on reasonable request.
