# Supplementary material for: Identification of new correctors for traffic-defective ABCB4 variants by a high-content screening approach
Source: Commun Biol. 2024 Jul 24;7:898. doi: 10.1038/s42003-024-06590-y (PMC11269752; doi:10.1038/s42003-024-06590-y)
Supplement: Supplementary file 1 — Supplementary Information [file 42003_2024_6590_MOESM1_ESM.pdf]

SUPPLEMENTARY INFORMATION

Identification of new correctors for traffic-defective ABCB4 variants by a high-content screening approach

Mounia Lakli[1,*], Julie Dumont[2,3,*], Virginie Vauthier[4], Julie Charton[2], Veronica Crespi[5], Manon Banet[1], Yosra Riahi[1], Amel Ben Saad[1], Elodie Mareux[1], Martine Lapalus[1], Emmanuel Gonzales[1,6], Emmanuel Jacquemin[1,6], Florent Di Meo[5], Benoit Deprez[2,3], Florence Leroux[2,3,§], Thomas Falguières[1,§,†]

[1] Inserm, Université Paris-Saclay, Physiopathogénèse et traitement des maladies du foie, UMR_S 1193, Hepatinov, F-91400 Orsay, France.

[2] Université de Lille, Inserm, Institut Pasteur de Lille, U1177 – Drugs and Molecules for Living Systems, F-59000 Lille, France.

[3] Université de Lille, CNRS, Inserm, CHU Lille, Institut Pasteur de Lille, US 41 - UAR 2014 - PLBS, F-59000 Lille, France.

[4] Inserm, Sorbonne Université, Centre de Recherche Saint-Antoine (CRSA), UMR_S 938, Institute of Cardiometabolism and Nutrition (ICAN), F-75012 Paris, France.

[5] Inserm, Université de Limoges, Pharmacology & Transplantation, UMR 1248, Centre de Biologie et Recherche en Santé, F-87000 Limoges, France.

[6] Assistance Publique - Hôpitaux de Paris, Paediatric Hepatology & Paediatric Liver Transplant Department, Reference Center for Rare Paediatric Liver Diseases, FILFOIE, ERN RARE LIVER, Faculté de Médecine Paris-Saclay, CHU Bicêtre, F-94270 Le Kremlin-Bicêtre, France.

* These authors contributed equally.

[§] These authors jointly supervised this work.

[†] **Corresponding author:** Thomas Falguières, PhD – UMR_S 1193 Inserm / Université Paris-Saclay – Bâtiment Henri Moissan – 17, avenue des Sciences – 91400 Orsay, France. Phone: +33-(0)1-69-15-62-94. e-mail: thomas.falguieres@inserm.fr

SUPPLEMENTARY METHODS

Immunoanalyses and measurement of ABCB4-mediated phosphatidylcholine secretion

After cell transfection and treatment, immunoblotting on HEK cell lysates was performed as described[1,2], using the following primary antibodies: anti-ABCB4, clone P3II-26 (Enzo Life Sciences, Villeurbanne, France) and anti-α-tubulin, clone 1E4C11 (ProteinTech, Manchester, UK). Proteins signals were detected by enhanced chemiluminescence using a Fusion Fx7 device (Vilber Lourmat, Collégien, France). Quantification of immunoblots was performed using ImageJ 1.50i software (U.S. National Institutes of Health, Bethesda, MD, USA). ABCB4 expression was normalized to the expression of α-tubulin.

Indirect immunofluorescence analyses were also performed as reported[1,2]. In brief, 48 hours post-transfection, ABCB4-expressing HepG2 cells grown on glass coverslips were fixed and permeabilized in ice-cold methanol (5 min on ice) and the following primary antibodies were used: anti-ABCB4 (clone P3II-26 – IgG2b; Enzo Life Sciences) and anti-ABCC2 (clone M2I4 – IgG1; Enzo Life Sciences). After incubation with isotype-specific secondary antibodies (anti-IgG2b-AlexaFluor™555 for ABCB4 and anti-IgG1-AlexaFluor™488 for ABCC2; Thermo Fisher Scientific, Villebon-sur-Yvette, France), nuclei were stained using Hoechst 33342 (Thermo Fisher Scientific) and coverslips were mounted on glass slides using Mowiol 4.88 (Merck, Saint-Quentin-Fallavier, France). Alternatively, to specifically detect plasma membrane mCherry-ABCB4-FLAG in HEK cells, primary anti-FLAG antibodies (F7425; Merck) were used in non-permeabilized cells as previously reported[2]. Immunofluorescence images were acquired with a confocal microscope (Eclipse TE-2000-Nikon-C2) equipped with a 60X 1.40 oil immersion objective. For quantification of ABCB4 localization at bile canaliculi, individual cells were analyzed for the colocalization of ABCB4 staining with the canalicular marker ABCC2, whether this colocalization was partial or total.

The measurement of ABCB4-mediated phosphatidylcholine (PC) secretion was already described[2,3]. Each tested condition was analyzed in triplicate and the amount of secreted PC was obtained after background subtraction. Then, final results were normalized to the expression levels of ABCB4 that were determined in parallel by immunoblot analyses of the corresponding cell lysates for each experiment.

Immunoanalyses in 384 well plates

Wells of 384-well plates were aspirated using a Bravo automated liquid handling platform (Agilent, Santa Clara, United States). Twenty microliters of rabbit anti-FLAG antibodies (Merck; 1:300 dilution) were added and plates were incubated at room temperature. After 1.5 hours, cells were washed four times with 65 µL phosphate-buffered saline (PBS) using a plate washer (Biotek EL406, Agilent, Santa Clara, CA, USA). Cells were fixed by adding 1% formaldehyde for 15 minutes and washed four times with PBS. Forty microliters of PBS containing 0.1% bovine serum albumin (BSA) were added. After 15 minutes, wells were emptied and 40 µL of a mixture of Hoescht 33342 (Thermo Fisher Scientific; 1 µg/mL) and anti-rabbit AlexaFluor^{TM}488-coupled secondary antibodies (Thermo Fisher Scientific; 1:400 dilution) in 0.05% BSA-containing PBS were added. After 1 hour, cells were washed eight times with a plate washer and plates were directly processed for image acquisition.

Chemical synthesis

For the synthesis of compound #2, 2-methyl-1-(4-piperidyl)-5-(trifluoromethyl)benzimidazole;hydrochloride (100 mg, 1 equiv.) and 2-methyl-1-[1-[[1-(2-thienylmethyl)tetrazol-5-yl]methyl]-4-piperidyl]-5-(trifluoromethyl)benzimidazole (74 mg, 1.1 equiv.) were stirred at room temperature in dichloromethane in presence of triethylamine (96 µL, 2.2 equiv.). After 24 hours and 72 hours, 40 µL (1 equiv.) of triethylamine were added. The reaction was complete after 7 days. The reaction mixture in dichloromethane was washed 3 times with H_2O. The organic phase was dried on $MgSO_4$ and concentrated under vacuum. The crude compound was purified by flash chromatography (CH_2Cl_2/MeOH) and by preparative HPLC. After lyophilization the compound was obtained as a white lyophilizate (67 mg, 46%). Purity (215 nM) = 100 %, MS [M + H]+ m/z =462. ^{1}H NMR (300 MHz, MeOD *d4*) 7.83 (s, 1H), 7.78 (d, *J*=8.7Hz, 1H), 7.50 (dd, *J*=8.8 Hz and 1.4 Hz, 1H), 7.49 (dd, *J*=5.1 Hz and 1.2 Hz, 1H), 7.24 (dd, *J*=3.4 Hz and 1.1 Hz, 1H), 7.07 (dd, *J*=5.1 Hz and 3.5 Hz, 1H), 6.02 (s, 2H), 4.49-4.41 (m, 1H), 3.98 (s, 2H), 3.02-2.99 (m, 2H), 2,68 (s, 3H), 2.54-2.39 (m, 4H), 1.93-1.89 (m, 2H). ^{13}C NMR (75 MHz, MeOD *d4*): 155.9, 153.7, 142.9, 137.6, 136.9, 129.1, 128.3, 128.0, 126.2 (q, *J*=271 Hz), 125.4 (q, *J*=31.7 Hz), 119.8 (q, *J*=3.6 Hz), 116.9 (q, *J*=4.1 Hz), 113.7, 55.7, 54.1; 51.1, 47.2, 30.9, 14.6.

Cell viability assays

Toxicity assessment of drug candidates was based on a colorimetric assay using the tetrazolium salt 3-(4,5-dimethylthiazol-2-yl)-2,5-diphenyltetrazolium bromide (MTT), which is reduced to an insoluble purple precipitate (formazan) by metabolically active cells[4], as published[2]. Briefly, HEK cells were seeded in a 96-well plate and treated with the candidate drugs at different concentrations (or DMSO as a control), including positive (1:5 DMSO) and negative (1:1,000 DMSO and no treatment) controls. After 16 hours of treatment, 100 µl of 0.25 mg/ml MTT (Merck, Saint-Quentin-Fallavier, France) was added to each well, and the cells were further incubated for 2 hours at 37°C. After incubation, culture media was washed out, and the cells were lysed in 100 µl of pure DMSO. Then, the absorbance at 550 nm, proportional to the number of alive cells in each well, was measured using a Wallac Victor^3 multilabel plate reader (PerkinElmer, Massy, France).

Preparation of ABCB4 models and ligands for molecular docking calculations

The unresolved extracellular loop of ABCB4^{if} and ABCB4^{cc} models was taken from our former study[2], and the linker connecting nucleotide binding domain 1 (NBD1) to transmembrane helix 7 (TMH7) was not included in our models. Likewise, unresolved N- and C-terminal regions were not considered in the present study. ABCB4 models were embedded in POPC:POPE:Chol (2:1:1) lipid bilayer and solvated in water with NaCl concentration at 0.154 M. ABCB4 models were minimized using a 4-step protocol: (i) water O-atom minimization, (ii) H-atom minimization, (iii) water minimization and (iv) whole system minimization. Ligands were initially optimized at the M06-2X/6-31+G(d,p) density functional theory to ensure proper bond distances and angles. This was achieved using the Gaussian16 software[5]. Frequency calculations were then carried out to ensure local minima by the absence of imaginary frequency.

Supplementary Figures

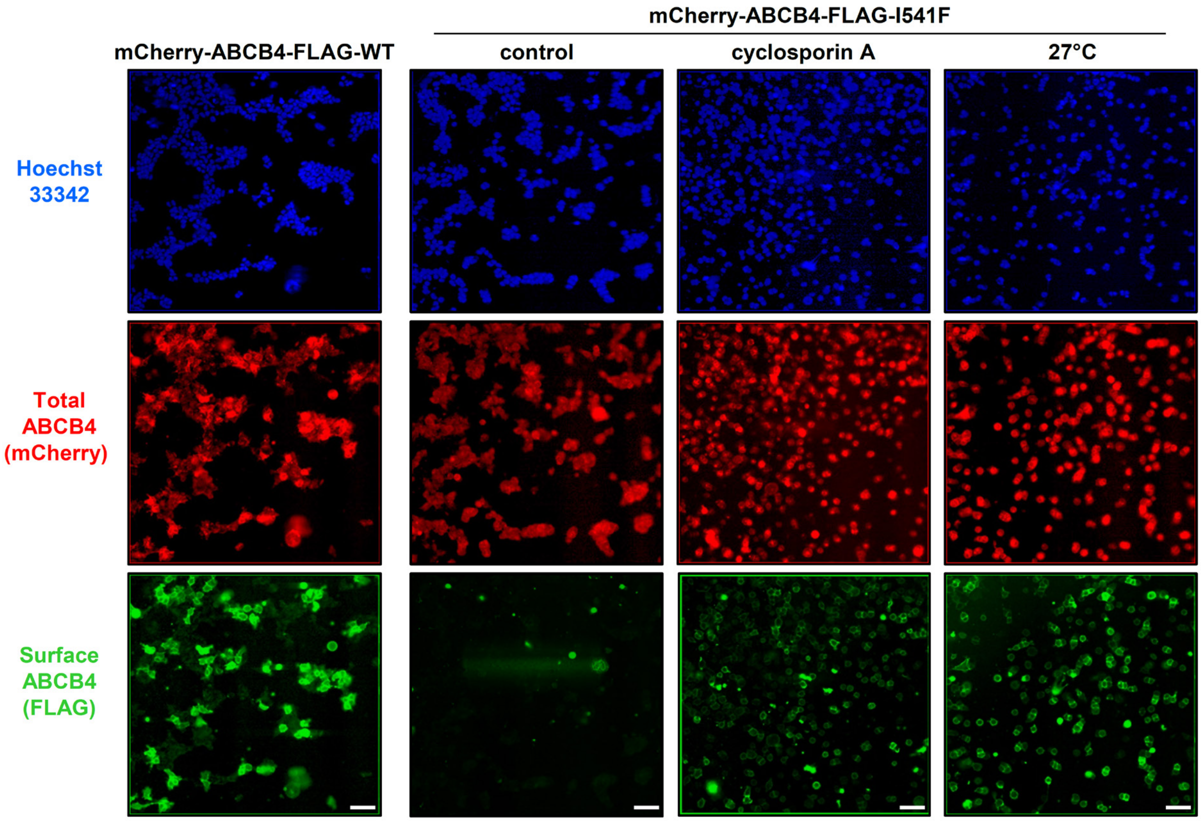

Supplementary Fig. 1 High-content screening imaging. HEK cells stably expressing mCherry-ABCB4-FLAG-WT or mCherry-ABCB4-FLAG-I541F were treated as in Fig. 1c and processed and imaged as described in Fig. 1d. Bars: 50 µm.

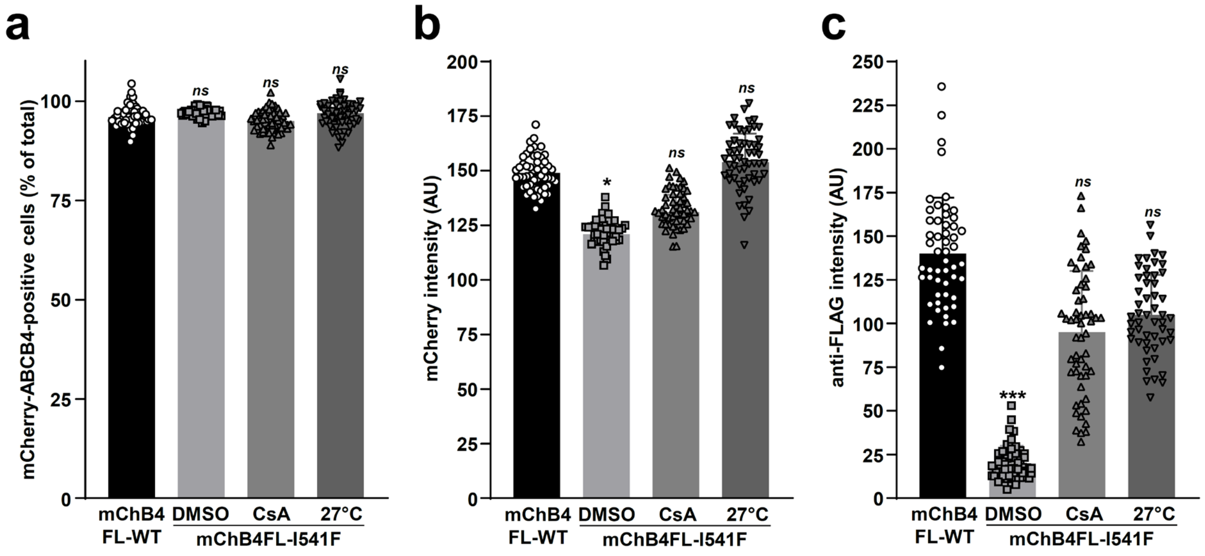

Supplementary Fig. 2 Quantifications for high-content screening set-up. a-c The percentage of mCherry-ABCB4-positive cells (a) and signal intensities of mCherry (b) and Alexa 488 (anti-FLAG antibodies) (c) were determined from automated analyses using Columbus^{TM} software. For each condition, means (± SD) of at least 48 independent wells (from 384-well plates) are represented. Cyclosporine A (CsA) was tested at 10 µM.

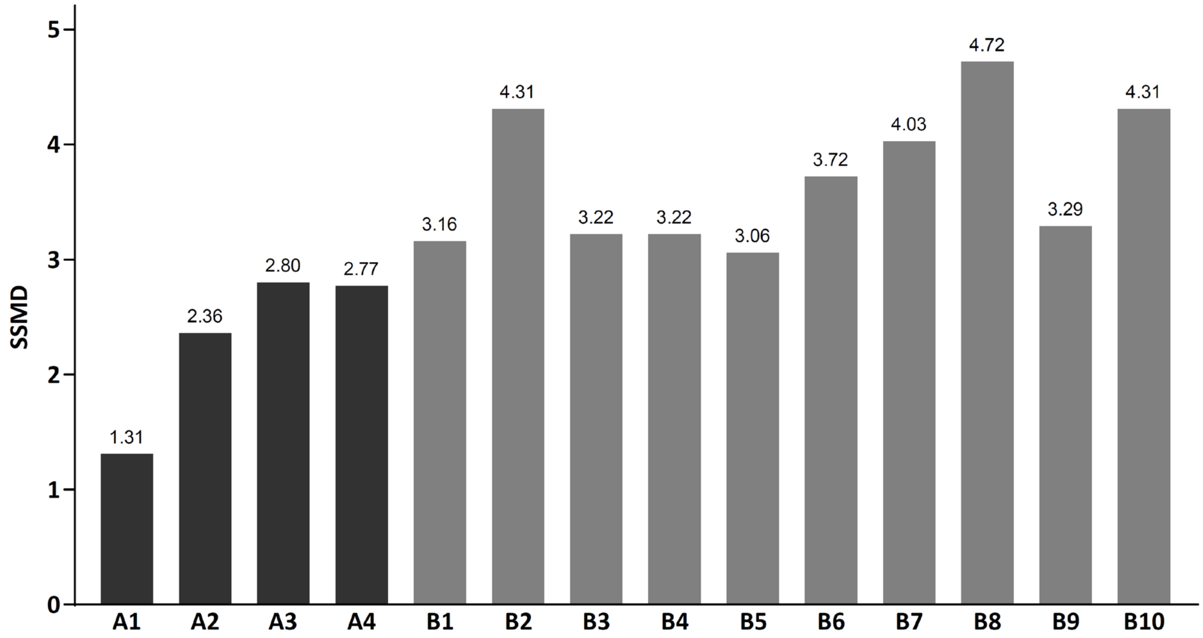

Supplementary Fig. 3 Compound screening in mCherry-ABCB4-FLAG-I541F-expressing HEK cells. Each 384-well plate was validated using strictly standardized mean difference (SSMD) values, calculated between DMSO-treated (controls) and 10 µM cyclosporin A (CsA)-treated conditions. The plates numbered A1 to A4 correspond to the Prestwick library, those numbered B1 to B10 to the second library. SSMD values were calculated using the mean µ and the variance σ of the distribution of Alexa 488 (anti-FLAG) signal intensities with the following equation:

$$SSMD = \frac{\mu_1 - \mu_2}{\sqrt{\sigma_1^2 + \sigma_2^2}}$$

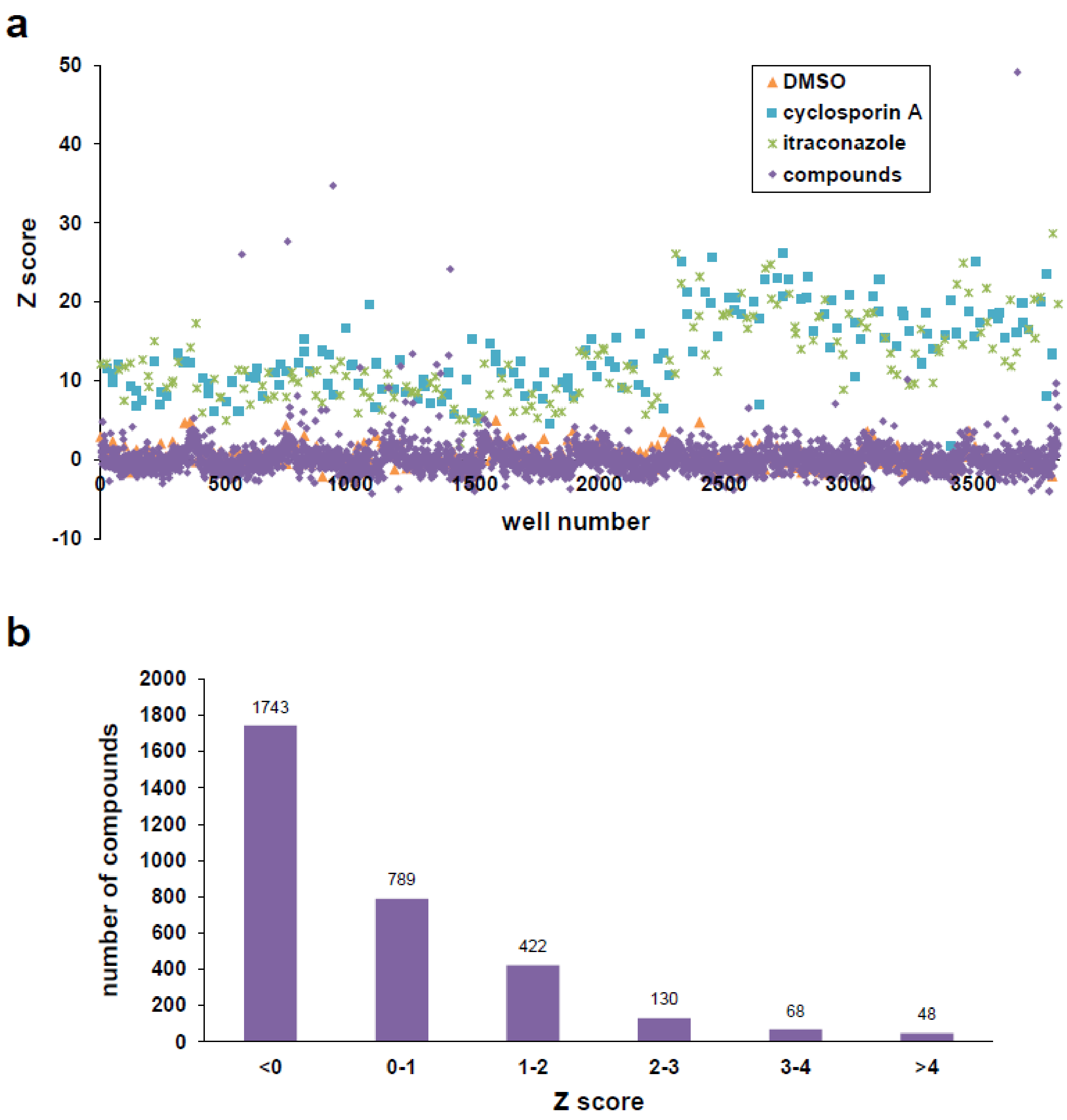

Supplementary Fig. 4 Z-score determination and distribution of the tested compounds. a Data plot of Z-scores for the drug/lead-like compound screening in mCherry-ABCB4-FLAG-I541F-expressing HEK cells. 3,200 compounds were tested at 10 µM. Cyclosporin and itraconazole were used at 10 µM as positive references. DMSO was used as a vehicle negative control. **b** Distribution of the compounds according to their Z-score (from A).

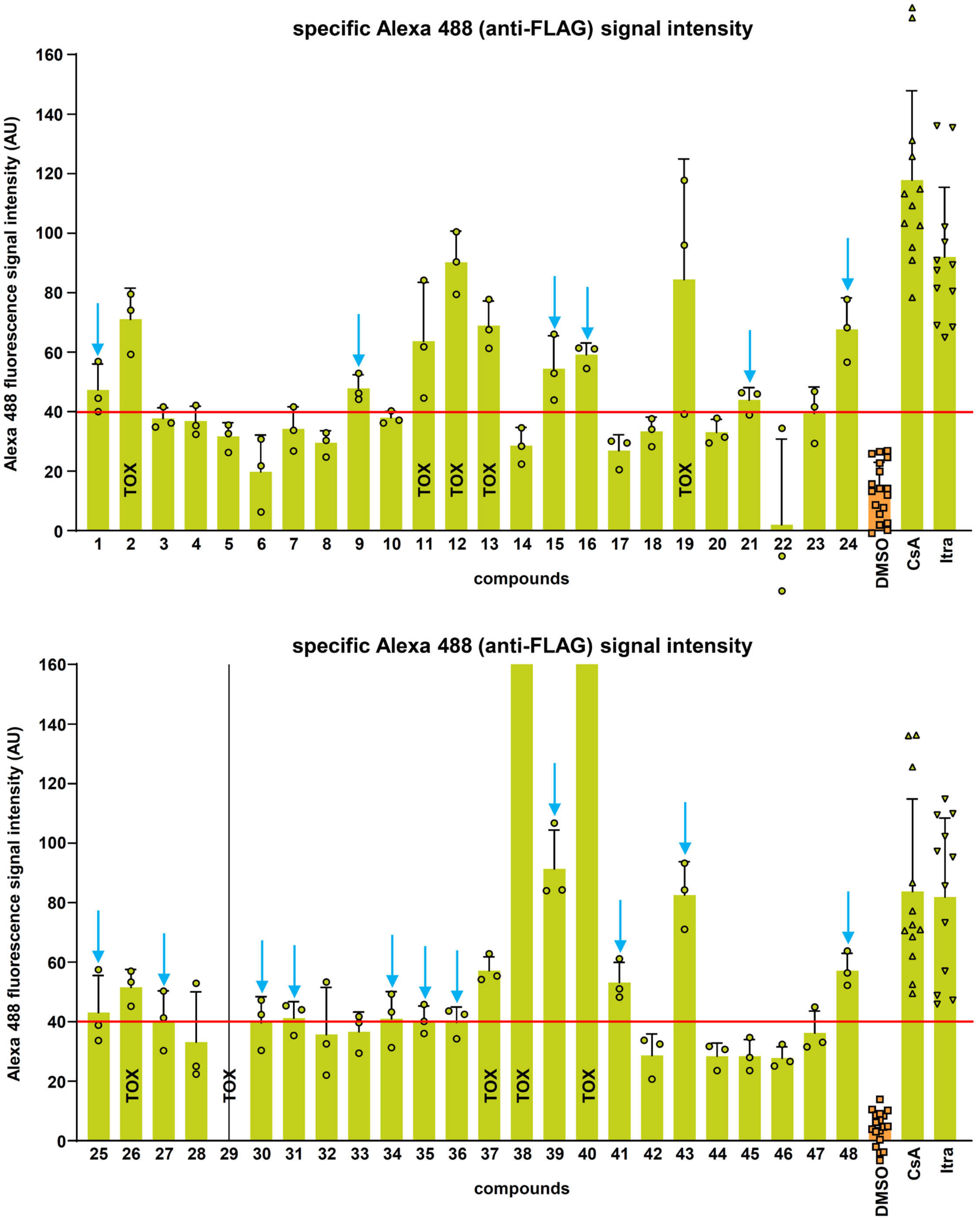

Supplementary Fig. 5 Drug/lead-like compound screening in mCherry-ABCB4-FLAG-I541F-expressing HEK cells. The selection of 17 compounds for dose-response curve experiments was based on the absence of toxicity and specific Alexa 488 (anti-FLAG) signal intensities. Each compound was tested at 10 µM and immunostaining was performed with or without anti-FLAG antibody. The difference of signal intensities between both treatments was calculated and the specific Alexa 488 (anti-FLAG) signal intensities were reported on this graph. “TOX” indicates toxicity of the compounds and blue arrows indicate the compounds selected to be further studied in dose-response curves experiments with a specific fluorescent threshold set at 40 (red line). Means (± SD) of three independent experiments are shown.

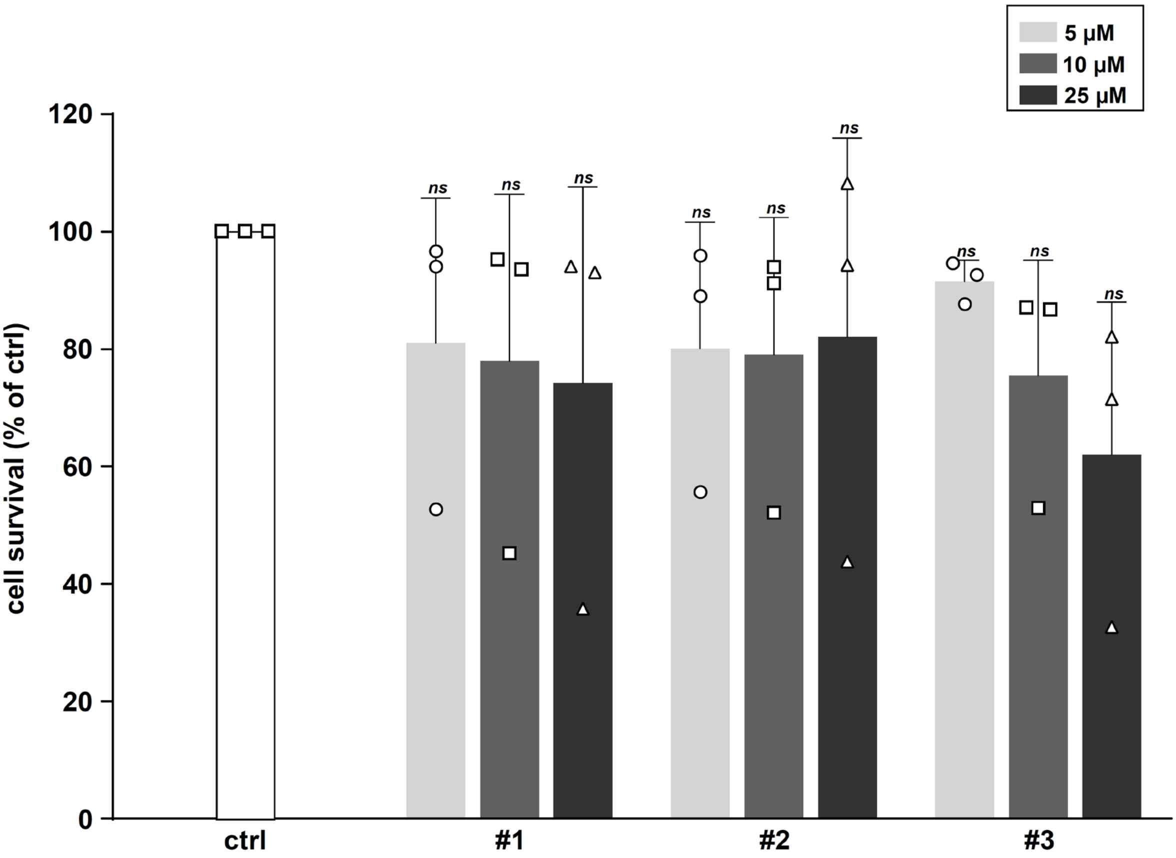

Supplementary Fig. 6 The HCS hits are not cytotoxic. HEK cells were treated with the indicated concentrations of hits or with the vehicle (ctrl, DMSO) for 16 hours. The cell viability was then assessed and expressed as the percentage of means for control vehicle-treated cells. Means (± SD) of at least three independent experiments per condition are shown.

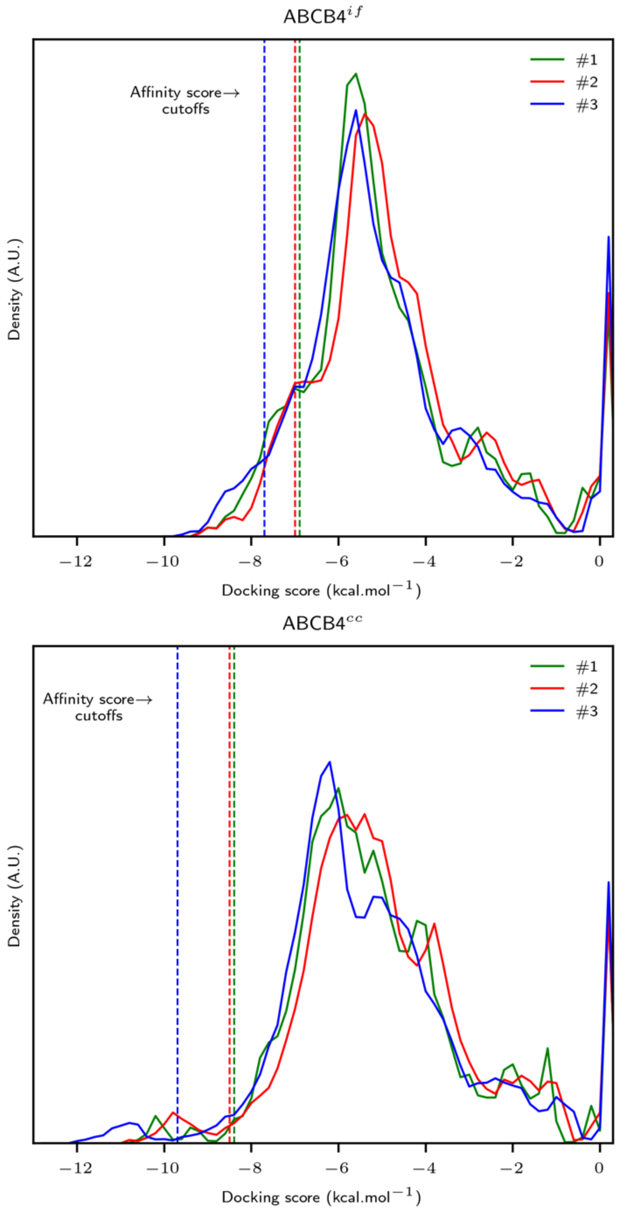

Supplementary Fig. 7 Distribution of docking affinity scores (kcal.mol^{-1}) obtained for compounds #1, #2 and #3, respectively depicted in green, red and blue. Applied affinity score cut-offs to select the most favorable poses are also shown as colored dashed lines.

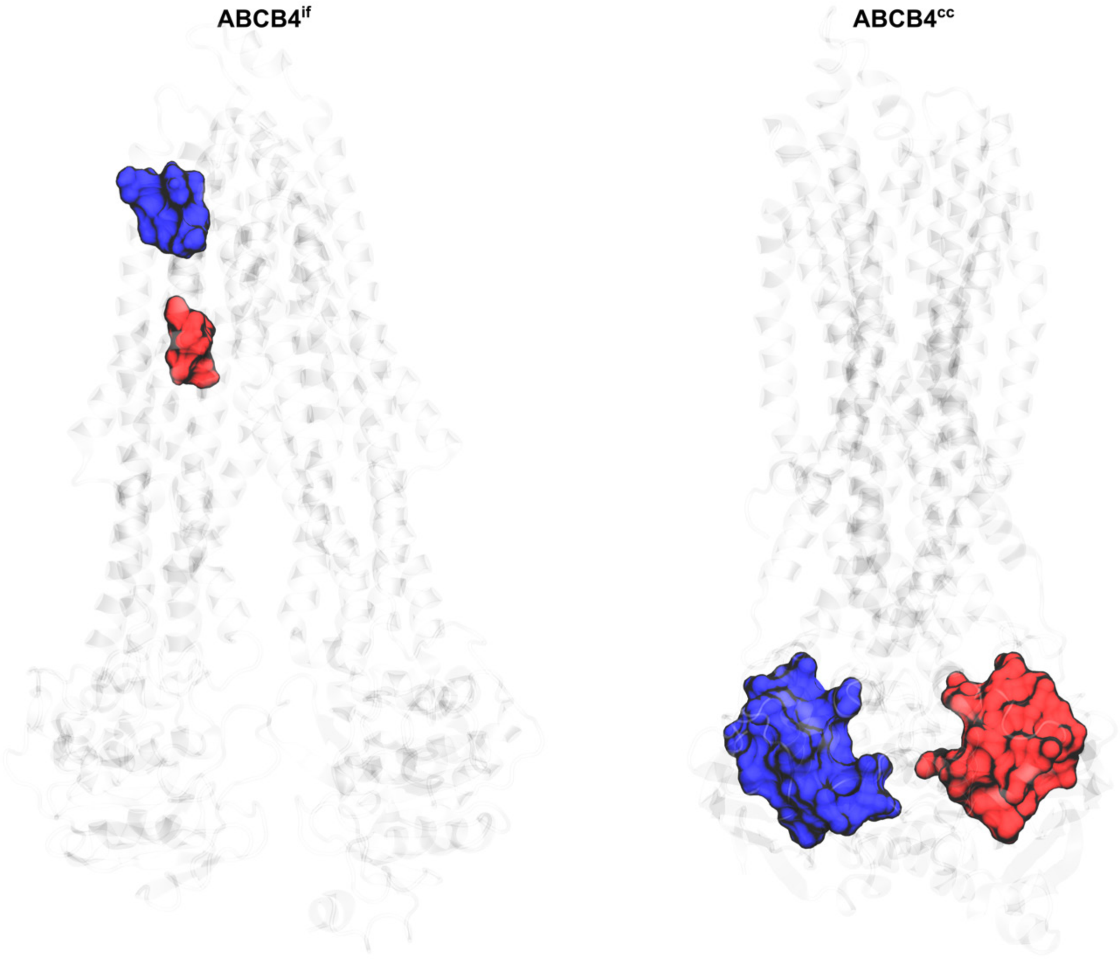

Supplementary Fig. 8 Predicted binding sites by PUResNET for ABCB4^{if} (left) and ABCB4^{cc} (right). The two predicted binding sites are color-coded in red and blue.

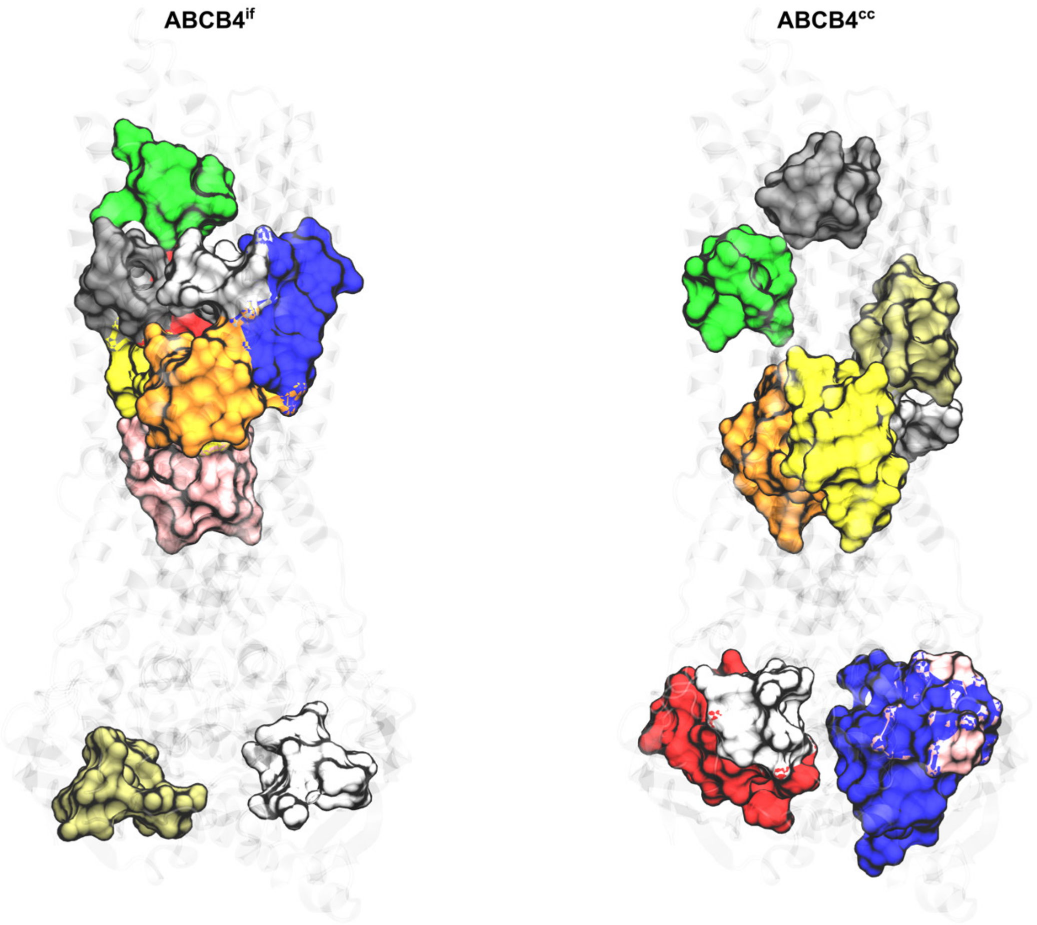

Supplementary Fig. 9 Predicted binding sites by PrankWEB for ABCB4^{if} (left) and ABCB4^{cc} (right). Residues, probabilities and color code are available in Supplementary Tables 6 & 7.

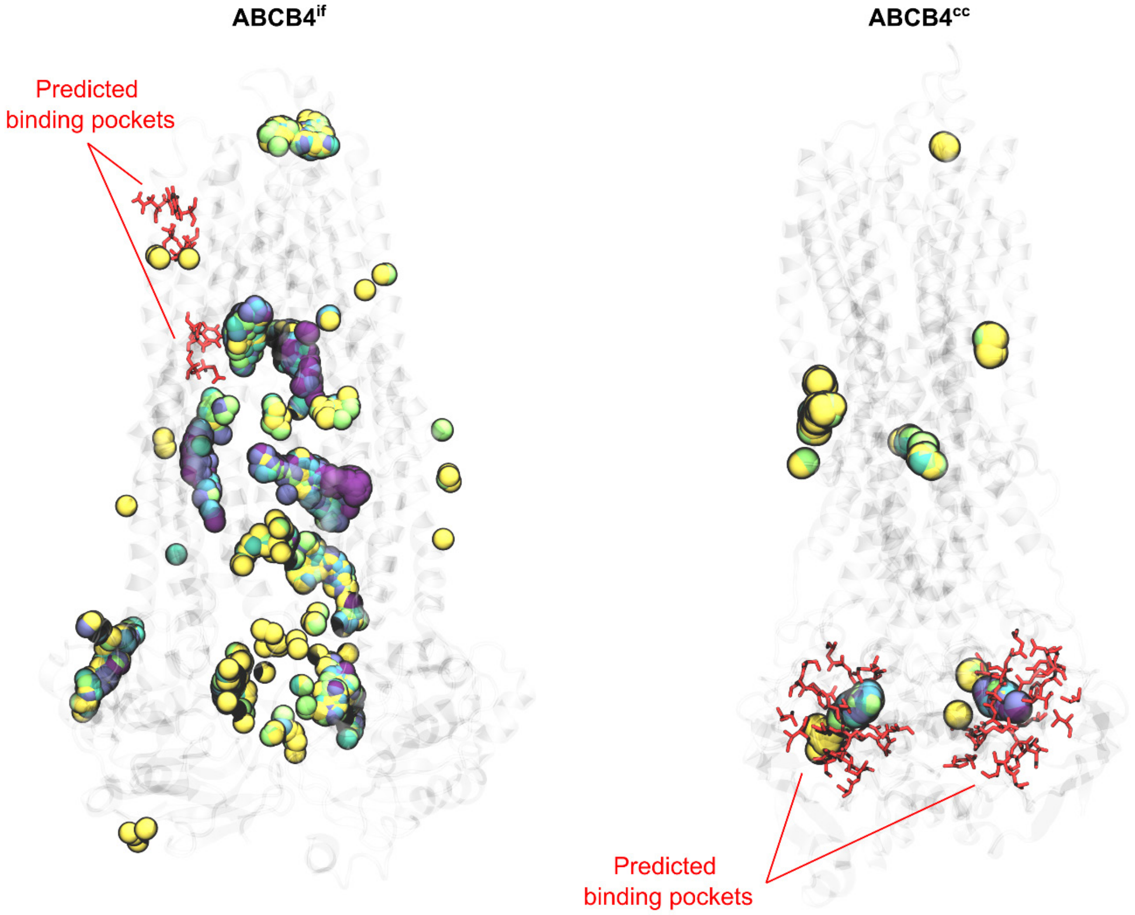

Supplementary Fig. 10 Comparison of molecular poses obtained from molecular docking calculations with binding sites predicted by PUResNET for ABCB4^{if} (left) and ABCB4^{cc} (right). The selected centers of mass for compounds #1, #2 and #3 were obtained from brute force molecular docking calculations for which cutoff of 2.5 kcal.mol^{-1} was applied. Centers of mass were colored considering the lowest score affinity at 0.0 kcal.mol^{-1}. Residues predicted to participate in predicted binding sites are depicted in red.

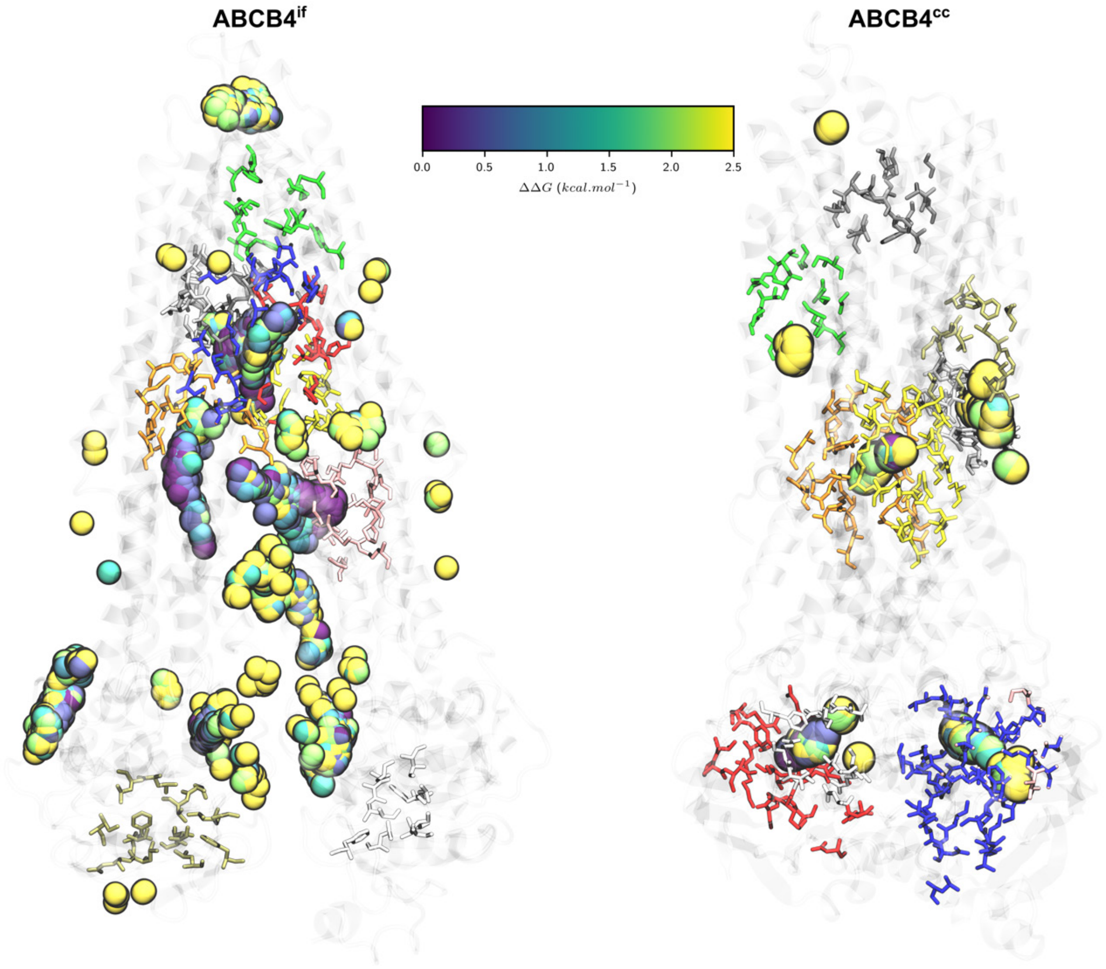

Supplementary Fig. 11 Comparison of molecular poses obtained from molecular docking calculations with binding sites predicted by PrankWEB for ABCB4^{if} (left) and ABCB4^{cc} (right). The selected centers of mass for compounds #1, #2 and #3 were obtained from brute force molecular docking calculations for which cutoff of 2.5 kcal.mol^{-1} was applied. Centers of mass were colored considering the lowest score affinity at 0.0 kcal.mol^{-1}. Residues predicted to participate in predicted binding sites are depicted according to predicted binding pocket color code proposed in Supplementary Tables 6 and 7.

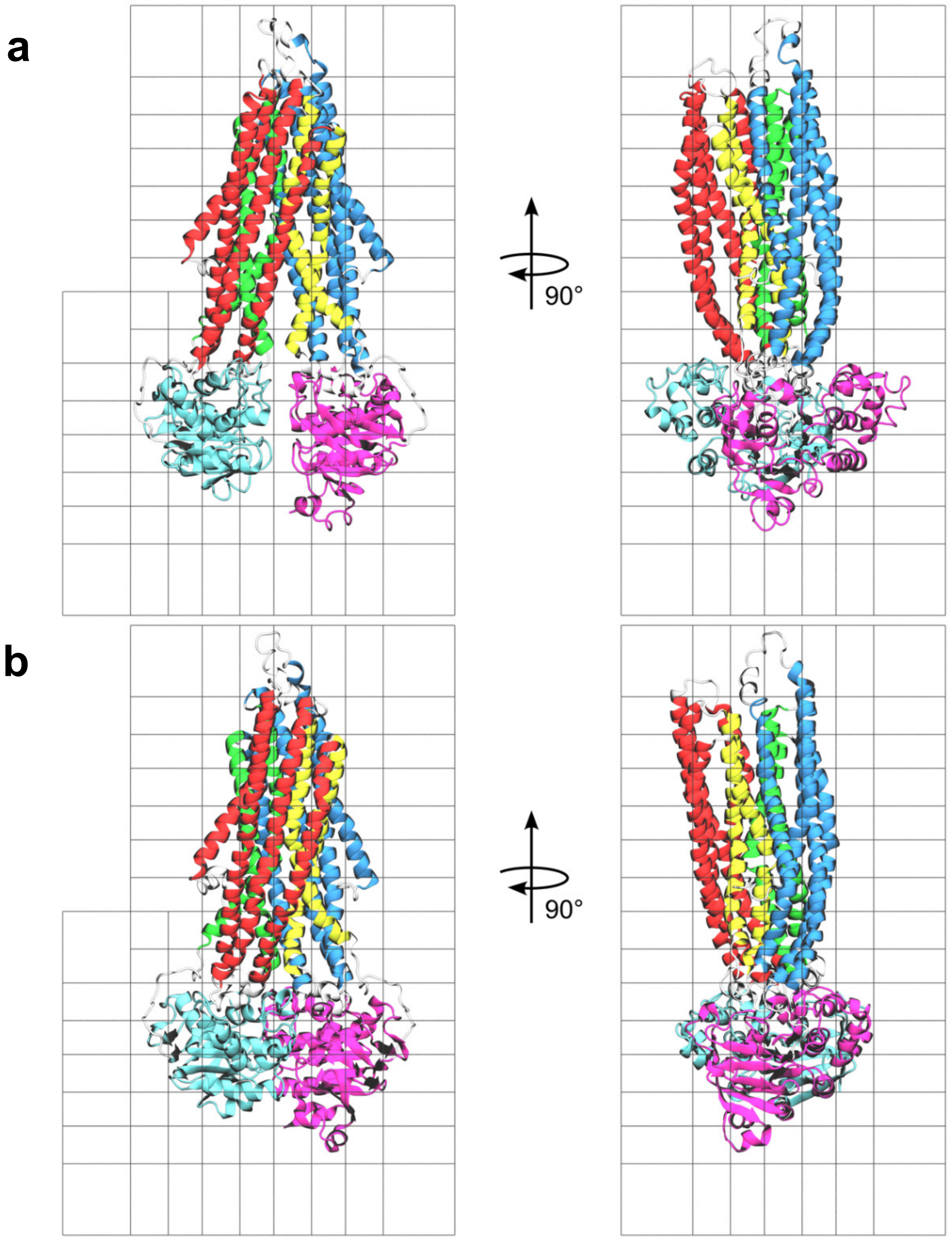

Supplementary Fig. 12 Molecular docking search volume boxes used for the brute force prediction of small molecule binding sites. a-b 113 sub-volumes were defined from ABCB4^{if} (a) and ABCB4^{cc} (b) protein structures to sample the whole molecular space considering all subdomains. Boxes distant to protein structure were kept as negative controls, for which no pose was obtained from molecular docking calculations.

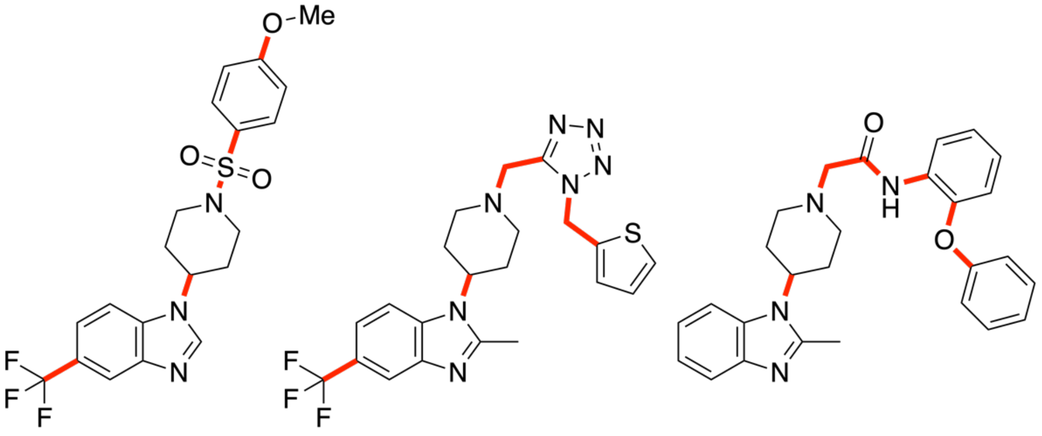

compound #1 compound #2 compound #3

Supplementary Fig. 13 Small compound rotatable dihedral angles for molecular docking calculations. Dihedral angle depicted in red were allowed to free rotate during molecular docking pose search.

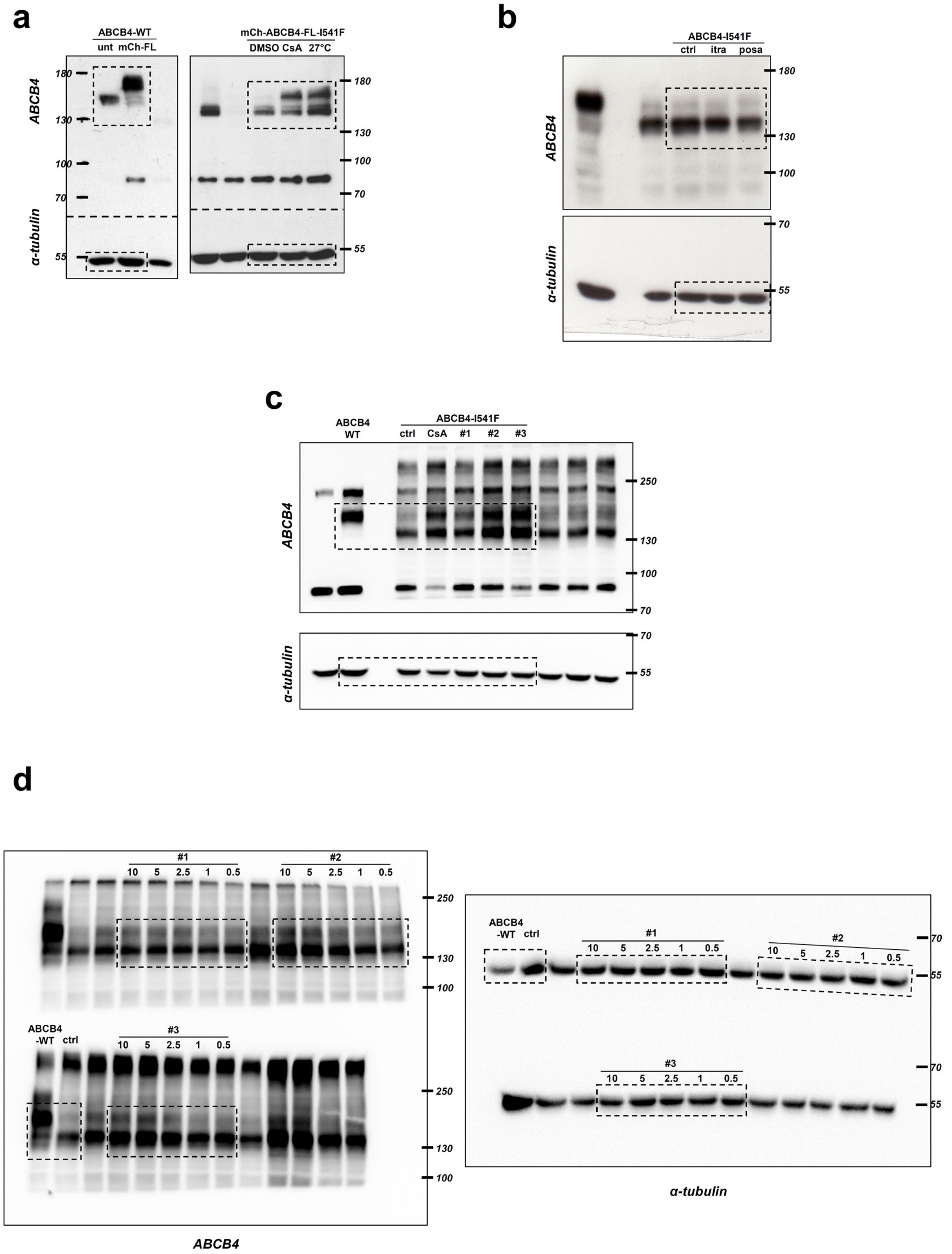

Supplementary Fig. 14 (first part)

e

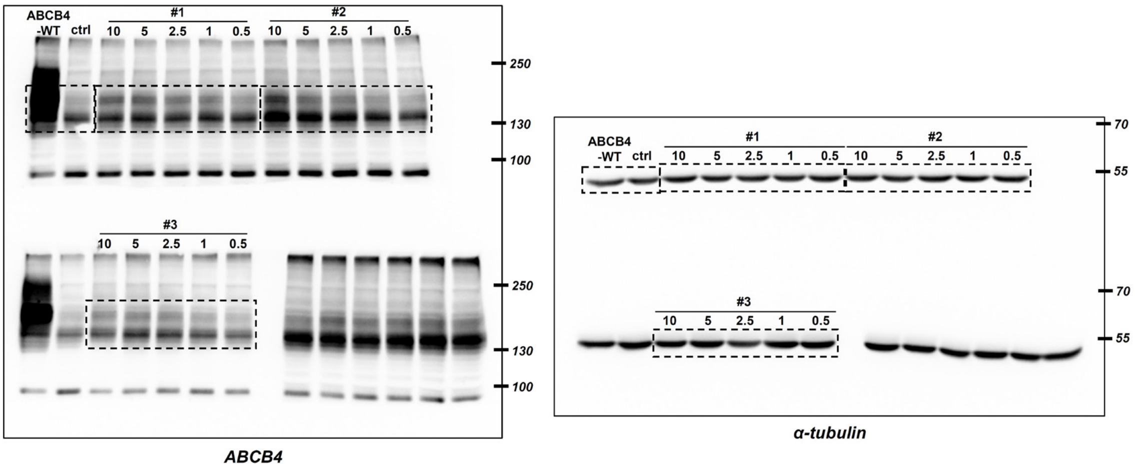

f

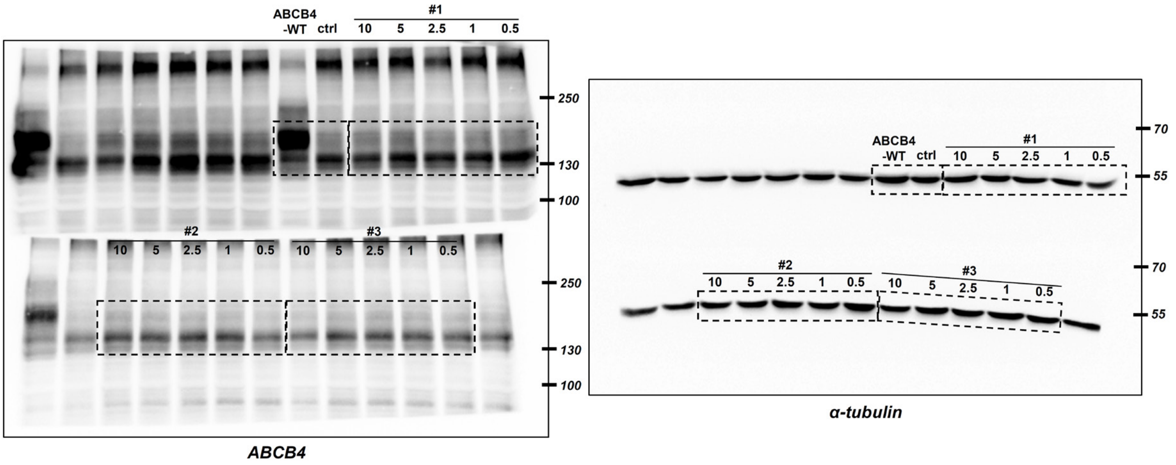

Supplementary Fig. 14 (second part)

Supplementary Fig. 14 Full immunoblots related to main figures, for Fig. 1a (a), Fig. 2d (b), Fig. 4a (c), Fig. 5c (d), Fig. 6a (e), Fig. 6c (f). Results shown in main figures are delineated by dotted rectangles. MW (in kDa) are indicated.

SUPPLEMENTARY TABLES

Supplementary Table 1 Compound screening in mCherry-ABCB4-FLAG-I541F-expressing HEK cells. Surface ABCB4 expression for vehicle and positive control incubates in both screening campaigns are indicated. Results are given as means ± SD.

	Surface ABCB4-positive cells (% of total ABCB4-expressing cells)	
	FDA-approved library	Drug/lead-like library
DMSO vehicle controls	7.5 ± 10.0 % (n=96)	7.0 ± 2.9 % (n=200)
10 µM CsA positive controls	46.9 ± 16.4 % (n=128)	47.5 ± 15.5 % (n=160)
10 µM itraconazole positive controls	not determined	43.3 ± 17.9 %(n=160)

Supplementary Table 2 Total number of poses obtained by molecular docking calculations for compounds #1, #2 and #3. The numbers of selected poses after applying an affinity score cut-off (2.5 $kcal.mol^{-1}$) are also reported. Docking Affinity score ($kcal.mol^{-1}$) ranges are also shown.

	$ABCB4^{if}$				$ABCB4^{cc}$			
Compounds	# Poses		Affinity Score		# Poses		Affinity Score	
	Total	Selected	Min.	Max.	# Poses	Selected	Min.	Max.
#1	44085	6455	-9.4	1.9	43849	325	-10.9	2.2
#2	44650	5156	-9.5	2.4	44397	1304	-11.0	1.9
#3	44718	3098	-10.2	1.9	44554	829	-12.2	2.1

Supplementary Table 3 Sequence-based topology of ABCB4 membrane transporter reporting residues located in transmembrane helices (TM) and nucleotide-binding domains (NBD), using the color code used in Fig. 8a-b.

	**Sequence**	**Color Code used in Fig. 8**
TM1	50-89	
TM2	104-159	
TM3	171-212	
TM4	215-261	
TM5	272-325	
TM6	330-372	
NBD1	392-629	
TM7	707-739	
TM8	746-798	
TM9	809-853	
TM10	856-901	
TM11	912-967	
TM12	969-1014	
NBD22	1033-1251	

Supplementary Table 4 Number of calculated atomic contact fractions per residue between compounds #1, #2 and #3 and ABCB4^{if} from molecular docking calculations. Contact analyses were performed over selected poses.

	#Residue	#1	#2	#3
TMH1	Ala89	1.13	0.78	-
ECL	Phe92	1.58	0.98	0.87
	Leu102	2.51	1.06	1.50
	Leu103	1.55	1.41	1.08
TMH2	Leu109	0.75	-	-
	Ile146	1.07	2.04	1.08
	Arg147	-	1.02	-
	Arg150	0.59	1.22	0.71
TMH3	Thr170	0.61	-	-
	Thr178	0.67	0.63	-
	Ser182	1.07	1.29	2.19
	Lys183	1.50	1.57	5.27
	Ser185	0.97	1.12	1.72
	Glu186	1.08	1.34	3.34
	Lys191	-	0.51	1.16
	Phe195	0.70	-	-
	Phe202	3.41	3.77	5.80
	Phe206	1.06	1.17	1.31
TMH4	Thr217	0.58	0.86	-
	Ile220	3.05	2.72	2.25
	Met221	-	0.67	-
	Ser224	2.56	2.58	4.18
	Leu227	5.49	4.99	7.10
	Gly228	-	-	1.03
	Ala231	2.02	1.73	2.37
	Trp234	2.87	1.83	2.40
	Tyr249	0.65	0.59	-
CpH	Arg264	1.74	1.85	1.48
	Gly270	1.63	2.19	-
TMH5	Gln272	-	0.56	-
	Asn273	-	0.88	-
	Leu283	-	0.50	-
	Lys287	0.70	0.78	-
	Gly290	0.71	0.72	-
	Lys293	-	1.17	-
	Ala294	-	1.16	-
	Asn298	-	0.65	-
	Met301	0.65	0.83	0.87
	Ala304	0.70	0.85	1.45
	Phe305	-	-	0.97
	Ile308	3.35	2.86	3.87
	Ser311	0.93	0.60	-
TMH6	Ile340	2.76	2.55	2.64
	Ala344	1.97	2.10	2.75
	Val347	2.47	2.30	2.44
	Gly348	1.30	1.34	1.57
	Gln349	-	0.69	-
	Ala351	-	0.64	0.72
	Ile354	1.26	0.78	0.94
	Ala356	0.71	0.70	1.31
	Phe357	0.88	-	-
	Asn359	-	-	1.70
NBD1	Val439	1.61	1.65	-
	Gln440	1.02	1.12	0.84
	Gln443	0.78	0.83	-
	Val474	0.62	0.89	-
	Ser476	1.40	1.48	0.86
	Gln477	1.99	2.19	1.61
	Glu478	0.62	0.59	0.89
	Val480	0.67	0.73	0.74
	Arg529	1.24	2.05	1.38

	#Residue	#1	#2	#3
TMH8	Gly773	-	0.93	-
	Phe776	-	0.60	-
	Gly777	0.86	1.37	0.87
	Glu781	1.32	1.30	0.72
	Thr784	1.01	1.00	1.05
TMH9	Thr815	0.72	-	-
	Arg816	0.84	-	-
	Thr819	0.97	-	-
	Gln825	0.61	0.71	0.99
	Thr830	-	0.72	0.72
	Ala833	-	0.72	-
	Leu834	-	0.65	-
	Leu842	2.06	2.55	4.34
	Ile846	1.45	1.49	2.02
TMH10	Leu861	1.00	1.18	1.61
	Val863	1.19	1.05	2.49
	Val864	1.38	1.81	3.05
	Ile867	1.50	1.67	3.03
	Ala868	-	0.80	1.06
	Leu878	-	0.75	2.06
	Asn881	-	-	0.72
	Ala882	1.28	1.69	4.01
	Asp885	0.79	0.70	1.32
	Lys886	0.96	1.02	0.77
	Leu889	0.80	0.63	-
	Glu890	0.79	0.70	-
	Thr897	-	0.61	-
	Glu901	-	0.64	-
CpH	Arg904	3.30	3.32	3.18
TMH11	Pro926	0.78	1.57	-
	Tyr927	0.57	1.15	-
	Ser930	0.95	1.38	0.96
	Lys933	1.39	2.05	4.30
	Met948	0.67	-	0.77
	Ile963	1.30	0.96	-
	Val964	0.94	0.70	-
TMH12	Arg969	1.51	0.56	1.06
	Ile980	1.14	1.77	1.78
	Ala984	1.04	0.90	2.05
	Leu987	1.42	1.70	3.27
	Ser991	-	0.55	0.89
	Phe993	-	-	1.19
	Pro995	1.59	1.74	4.13
	Asp996	-	0.50	1.65
	Lys999	1.50	1.42	2.97
ICD	Ser1019	-	1.04	-
	Tyr1020	1.35	1.28	0.73
NBD2	Val1079	-	0.58	-
	Gln1080	0.84	0.75	-
	Gln1106	2.44	3.10	0.88
	Trp1107	-	0.96	-
	Arg1109	-	0.80	-
	Ala1110	2.42	2.82	-
	Gln1111	-	0.77	-
	Ile1114	0.71	0.71	-
	Ser1116	0.93	0.71	-
	Gln1117	1.34	1.33	0.87
	Glu1118	0.74	-	-
	Asp1134	0.65	0.76	-
	Asn1135	1.62	1.53	-
	Ser1136	1.87	2.12	-
	Lys1171	0.66	0.69	-

Supplementary Table 5 Number of calculated atomic contact fractions per residue between compounds #1, #2 and #3 and ABCB4^{cc} from molecular docking calculations. Contact analyses were performed over selected poses.

	Residue	#1	#2	#3
TMH2	Thr139	-	1.52	-
	Gly143	-	1.37	-
	Ile146	-	2.00	-
	Arg147	-	1.78	-
	Arg150	-	2.66	-
TMH3	Ser185	-	1.16	-
	Glu186	-	7.00	-
	Asp190	-	1.55	-
	Lys191	-	5.10	-
	Phe195	-	-	3.14
	Arg264	13.42	3.38	9.04
TMH5	Lys293	-	2.73	-
	Ala294	-	2.23	-
	Ala297	-	1.19	-
TMH6	Ile354	-	-	2.96
	Asp355	-	2.04	-
	Ala356	-	2.53	-
	Phe357	-	-	4.98
	Asn359	-	2.17	-
NBD1	Tyr403	3.01	9.24	17.42
	Arg406	-	5.63	3.76
	Val409	-	1.82	-
	Ile411	-	5.84	3.04
	Ser436	-	3.39	4.14
	Thr437	-	1.56	2.90
	Gln440	4.09	8.04	9.92
	Ser476	-	2.46	3.98
	Gln477	-	3.96	4.22
	Glu478	-	2.31	-
	Val480	-	2.85	4.17
	Leu518	-	2.11	-
	Arg529	10.73	2.86	4.96
	Ala531	11.68	2.26	6.09
	Gln532	15.62	2.24	13.18
	Leu533	6.08	2.02	4.14
	Ser534	17.14	5.27	9.71
	Lys538	3.59	-	-

	Residue	#1	#2	#3
TMH9	Gln825	-	1.85	-
	Gly826	-	3.92	-
	Thr830	-	3.10	-
	Arg831	-	4.55	-
	Leu842	-	1.06	-
TMH10	Val864	-	1.00	-
	Arg904	3.22	8.25	15.24
	Thr905	-	-	2.26
TMH11	Pro926	-	1.23	-
	Tyr927	-	1.71	-
	Ser930	-	2.18	-
	Lys933	-	2.26	-
TMH12	Leu987	-	1.41	-
	Pro995	-	1.91	-
	Asp996	-	5.18	-
	Lys999	-	5.04	-
NBD2	Tyr1043	28.11	8.32	16.66
	Arg1046	20.53	6.63	4.94
	Val1049	-	2.33	-
	Val1051	-	3.08	2.91
	Ser1076	8.87	1.62	3.09
	Thr1077	4.29	1.06	2.38
	Gln1080	9.55	3.99	5.97
	Ser1116	-	1.42	2.93
	Gln1117	6.79	3.27	2.14
	Glu1118	7.32	4.80	4.35
	Ile1120	7.29	1.97	2.75
	Phe1156	-	1.36	-
	Thr1173	-	3.64	6.63
	Gln1174	-	2.05	12.39
	Leu1175	-	2.47	4.79
	Ser1176	3.34	9.16	13.62
	Gln1179	-	1.26	-
	Lys1180	-	1.09	-

Supplementary Table 6 Predicted binding pockets of ABCB4^{if} obtained from PrankWEB online webserver, including color code for Supplementary Figures 9 & 11. Predicted binding pocket with a probability lower than 0.1 were not considered for comparison with molecular docking calculations.

Rank	Score	Probability	Residues
1	21.84	0.857	Phe202, Gly205, Phe206, Thr217, Ile220, Met221, Ser224, Leu227, Ser230, Ala231, Trp234, Ser300, Met301, Ala304, Phe305, Ile308, Ser311, Tyr312, Ser339, Ile340, Ala344, Val347, Gly348
2	10.03	0.582	Met194, Phe195, Gln197, Ala198, Thr201, Ile342, Ser346, Gln349, Ala350, Cys353, His67, Leu71, Phe941, Ser944, Gln945, Met948, Tyr949, Val981, Ala984, Val985, Leu987, Gly988
3	9.96	0.578	Leu842, Ile846, Ser849, Leu860, Leu861, Val863, Val864, Ile867, Ser944, Met948, Ile980, Gly983, Ala984, Leu987
4	8.3	0.488	Trp234, Ala294, Ala297, Asn298, Met301, Phe305, Gly348, Gln349, Ala351, Pro352, Asp355, Phe769, Gln772, Gly773, Phe776, Gly777, Gly829, Thr830, Ala833, Leu834, Gln837
5	6.55	0.36	Glu186, Asp190, Lys191, Met194, Ile867, Gly871, Glu874, Tyr937, Thr940, Phe941, Ser944, Leu987, Gly988, Ser991, Ser992
6	6.12	0.328	Ile1034, Phe1036, Leu1052, Leu1055, Leu1057, Glu1058, Leu1067, Gly1074, Thr1077, Val1078, Leu1081, Leu1082, Ile1227, Ile1229, Phe1245
7	5.44	0.272	Phe305, Ile308, Tyr309, Tyr312, Leu341, Phe345, Leu724, Gln725, Phe728
8	4.31	0.192	Met113, Tyr120, Phe78, Met81, Leu856, Phe956, Ile963, Phe970, Val973, Ile974, Phe977
9	3.73	0.149	Phe396, Leu415, Leu417, Leu427, Thr438, Val439, Ile442, Leu555, Ile585, Phe603
10	3.56	0.137	Thr139, Ala142, Gly143, Ile146, Arg150, Ile181, Ser182, Ser185, Glu186, Leu878, Asn881, Ala882, Asp885, Leu889, Pro926, Asn929, Ser930, Lys933
11	*2.31*	*0.059*	*Glu245, Tyr249, Leu283, Ala286, Lys287, Gly290, Gly780, Glu781, Thr784, Arg788, Ala822, Gln825*
12	*1.98*	*0.041*	*Tyr403, Ser431, Gly432, Lys435, Ser436, Thr437, Gln440, Gln477, Gln558*
13	*1.78*	*0.032*	*Lys191, Pro352, Asp355, Ala356, Asn359, Gly826, Thr830, Ser992, Phe993, Asp996*

Supplementary Table 7 Predicted binding pockets of ABCB4^{cc} obtained from PrankWEB online webserver, including color code for Supplementary Figures 9 & 11. Predicted binding pocket with a probability lower than 0.1 were not considered for comparison with molecular docking calculations.

Rank	Score	Probability	Residues
1	36.76	0.947	Phe1156, Leu1160, Thr1173, Gln1174, Leu1175, Ser1176, Gly1177, Gly1178, Gln1179, Lys1180, Ala1204, Leu1205, Asp1206, Tyr403, Arg406, Val409, Ile411, Leu427, Val428, Gly429, Ser430, Ser431, Gly432, Cys433, Gly434, Lys435, Ser436, Thr437, Val439, Gln440, Gln443, Val474, Ser476, Gln477, Glu478, Asp557, Gln558, Ile587, Ala588, Leu591, Arg904, Thr905, Ser908
2	10.45	0.601	Tyr1043, Arg1046, Val1049, Pro1050, Val1051, Ser1071, Gly1072, Cys1073, Gly1074, Lys1075, Ser1076, Thr1077, Gln1080, Gln1117, Asp1199, Gln1200, Phe514, Lys517, Leu518, Ala531, Gln532, Leu533, Ser534, Gly535, Gln537, Ala562, Asp564
3	8.83	0.519	Gly331, Met334, Thr335, Phe338, Ser729, Phe732, Ser733, Ile736, Met75, Phe78, Thr82, Ile974, Leu975, Ser978
4	8.31	0.488	Thr139, Ala142, Gly143, Ile146, Arg150, Ile181, Ser182, Ser185, Glu186, Gly189, Asp190, Lys191, Gln823, Leu878, Asn881, Ala882, Asp885, Leu889, Pro926, Asn929, Ser930, Lys933, Tyr937, Ser992, Pro995, Asp996, Lys999
5	8.28	0.486	Asp179, Lys183, Glu186, Ser242, Glu245, Leu246, Tyr249, Gly290, Lys293, Ala294, Ala297, Asn298, Pro352, Asp355, Ala356, Asn359, Gly777, Glu781, Gln825, Gly826, Gly829, Thr830, Arg831, Leu834
6	6.48	0.355	Phe195, Val199, Phe202, Phe203, Ser224, Leu227, Gly228, Ser230, Ala231, Ala232, Ser300, Ala304, Val347, Gly348, Ala351, Ile354
7	4.9	0.23	Trp138, Val192, Phe195, Phe196, Cys353, Ile354, Phe357, Val42, Leu43, Phe46, Ser49, Lys54, Met57, Ser58, Thr61
8	3.64	0.143	Leu842, Ile846, Leu860, Leu861, Val863, Val864, Ile867, Met948, Ser951, Ile980, Gly983, Leu987
9	3.08	0.105	Ser1076, Val1079, Gln1080, Phe1085, Ile1114, Ser1116, Gln1117, Asp1199, Arg264, Thr265, Ala268, Ala531
10	3.05	0.103	Phe1156, Thr1159, Leu1160, Gln1174, Leu1175, Gln1179, Tyr403, Ser405, Arg406, Val409, Ile411, Gly432
11	*2.75*	*0.084*	*Phe202, Ile220, Ile223, Leu227, Ile308, Ser311, Tyr312, Ala344*
12	*2.24*	*0.055*	*His1006, Met1009, Leu1010, Arg1013, Trp802, Lys807, Leu813, Arg816, Asp820*
13	*2.19*	*0.052*	*Gln197, Thr201, Ser346, His67, Gly68, Leu71, Gln945, Tyr949, Ala984, Val985*
14	*1.92*	*0.038*	*Ala559, Thr560, Gln572, Val586, Ile587, Ala588, His589, Arg590, Thr593*
15	*1.25*	*0.013*	*Gly319, Val323, Ile330, Met334, Phe337, Phe739*
16	*1.09*	*0.009*	*Phe697, Leu832, Ile835, Ile839, Phe993, Tyr997*
17	*0.85*	*0.004*	*Phe1030, Ile1034, Leu1095, Leu1108, Gln1111, Leu1112, Ile1195*
18	*0.63*	*0.001*	*Ile731, Phe754, Ile757, Phe758, Leu761*

SUPPLEMENTARY REFERENCES

1 Vauthier, V. et al. Structural analogues of roscovitine rescue the intracellular traffic and the function of ER-retained ABCB4 variants in cell models. *Sci Rep* **9**, 6653 (2019).

2 Ben Saad, A. et al. Effect of CFTR correctors on the traffic and the function of intracellularly retained ABCB4 variants. *Liver Int* **41**, 1344-1357 (2021).

3 Gautherot, J. et al. Phosphorylation of ABCB4 impacts its function: Insights from disease-causing mutations. *Hepatology* **60**, 610-621 (2014).

4 van Meerloo, J., Kaspers, G. J. & Cloos, J. Cell sensitivity assays: the MTT assay. *Methods Mol Biol* **731**, 237-245 (2011).

5 Frisch, M. J. et al. Gaussian 16 Rev. C.01 (Wallingford, CT, 2016).
