# Supplementary material for: Identification of new correctors for traffic-defective ABCB4 variants by a high-content screening approach
Source: Commun Biol. 2024 Jul 24;7:898. doi: 10.1038/s42003-024-06590-y (PMC11269752; doi:10.1038/s42003-024-06590-y)
Supplement: Supplementary file 2 — Description of Additional Supplementary Materials [file 42003_2024_6590_MOESM2_ESM.pdf]

Description of Additional Supplementary Files

File name: Supplementary Data 1

Description: Data tables supporting graphs provided in this study
