# Supplementary material for: Identification of new correctors for traffic-defective ABCB4 variants by a high-content screening approach
Source: Commun Biol. 2024 Jul 24;7:898. doi: 10.1038/s42003-024-06590-y (PMC11269752; doi:10.1038/s42003-024-06590-y)
Supplement: Supplementary file 4 — Reporting Summary [file 42003_2024_6590_MOESM4_ESM.pdf]

nature portfolio

Corresponding author(s): DAPR COMMSBIO-24-0780

Last updated by author(s): Mar 16, 2024

Reporting Summary

Nature Portfolio wishes to improve the reproducibility of the work that we publish. This form provides structure for consistency and transparency in reporting. For further information on Nature Portfolio policies, see our Editorial Policies and the Editorial Policy Checklist.

Statistics

For all statistical analyses, confirm that the following items are present in the figure legend, table legend, main text, or Methods section.

n/a	Confirmed	
☐	☒	The exact sample size (*n*) for each experimental group/condition, given as a discrete number and unit of measurement
☐	☒	A statement on whether measurements were taken from distinct samples or whether the same sample was measured repeatedly
☐	☒	The statistical test(s) used AND whether they are one- or two-sided<br>*Only common tests should be described solely by name; describe more complex techniques in the Methods section.*
☒	☐	A description of all covariates tested
☒	☐	A description of any assumptions or corrections, such as tests of normality and adjustment for multiple comparisons
☐	☒	A full description of the statistical parameters including central tendency (e.g. means) or other basic estimates (e.g. regression coefficient) AND variation (e.g. standard deviation) or associated estimates of uncertainty (e.g. confidence intervals)
☒	☐	For null hypothesis testing, the test statistic (e.g. *F*, *t*, *r*) with confidence intervals, effect sizes, degrees of freedom and *P* value noted<br>*Give P values as exact values whenever suitable.*
☒	☐	For Bayesian analysis, information on the choice of priors and Markov chain Monte Carlo settings
☒	☐	For hierarchical and complex designs, identification of the appropriate level for tests and full reporting of outcomes
☒	☐	Estimates of effect sizes (e.g. Cohen's *d*, Pearson's *r*), indicating how they were calculated

Our web collection on statistics for biologists contains articles on many of the points above.

Software and code

Policy information about availability of computer code

Data collection	The following software were used for data collection: In cell analyzer version 1.0 (GE Healthcare Life Sciences, Buc, France); NIS-Elements version AR 4.50 (Nikon Instruments Inc, Melville, NY, USA); Fusion version 15.11 (Vilbert Lourmat, Collégien, France); i-control version 2.0 (Tecan, Männedorf, Switzerland); CHARMM-GUI webserver (https://www.charmm-gui.org/); AMBERTools suite version 18 (University of California, CA, USA).
Data analysis	Data were analyzed using: Columbus software version 2.9.1 (Perkin Elmer Informatics, Waltham, MA, USA); Prism version 9.5.1 (GraphPad software, Boston, MA, USA); NIS-Elements version AR 4.50 (Nikon Instruments Inc, Melville, NY, USA); Photoshop version 8.0.1 (Adobe, San José, CA, USA); ImageJ version 1.53t (NIH, Bethesda, MD, USA); AutoDock Vina version 1.2.5 (The Scripps Research Institute, La Jolla, CA, USA); Vina-GPU software version 2.0 (https://github.com/DeltaGroupNJUPT/Vina-GPU-2.0); CPPTRAJ (DOI: 10.1021/ct400341p), PyTRAJ19 (https://github.com/Amber-MD/pytraj); VMD versions 1.9.3 and 1.9.4 (https://www.ks.uiuc.edu/Research/vmd/) and ChemDraw version 12.0.2 (CambridgeSoft, Cambridge, MA, USA).

For manuscripts utilizing custom algorithms or software that are central to the research but not yet described in published literature, software must be made available to editors and reviewers. We strongly encourage code deposition in a community repository (e.g. GitHub). See the Nature Portfolio guidelines for submitting code & software for further information.

Data

Policy information about availability of data

All manuscripts must include a data availability statement. This statement should provide the following information, where applicable:

- Accession codes, unique identifiers, or web links for publicly available datasets
- A description of any restrictions on data availability
- For clinical datasets or third party data, please ensure that the statement adheres to our policy

There is no restriction on data availability and no data with mandated deposition. The datasets generated and analyzed during this study are available from the authors on reasonable request.

Research involving human participants, their data, or biological material

Policy information about studies with human participants or human data. See also policy information about sex, gender (identity/presentation), and sexual orientation and race, ethnicity and racism.

Reporting on sex and gender	Not applicable
Reporting on race, ethnicity, or other socially relevant groupings	Not applicable
Population characteristics	Not applicable
Recruitment	Not applicable
Ethics oversight	Not applicable

Note that full information on the approval of the study protocol must also be provided in the manuscript.

Field-specific reporting

Please select the one below that is the best fit for your research. If you are not sure, read the appropriate sections before making your selection.

☒ Life sciences ☐ Behavioural & social sciences ☐ Ecological, evolutionary & environmental sciences

For a reference copy of the document with all sections, see nature.com/documents/nr-reporting-summary-flat.pdf

Life sciences study design

All studies must disclose on these points even when the disclosure is negative.

Sample size	No sample-size calculation was performed ahead of experiments. Each individual condition was tested at least three independent times (see figure legends) in order to fullfil statistical requirements.
Data exclusions	No data were excluded.
Replication	The number of experimental replicates is indicated in the figure legends.
Randomization	No randomization was applied to this study.
Blinding	Blinding was not applied to this study since experiments were mostly interpreted by people who performed them or were aware of experimental conditions. However, particular attention was paid to ensuring that results were interpreted with impartiality and integrity.

Reporting for specific materials, systems and methods

We require information from authors about some types of materials, experimental systems and methods used in many studies. Here, indicate whether each material, system or method listed is relevant to your study. If you are not sure if a list item applies to your research, read the appropriate section before selecting a response.

Materials & experimental systems

n/a	Involved in the study
☐	☒ Antibodies
☐	☒ Eukaryotic cell lines
☒	☐ Palaeontology and archaeology
☒	☐ Animals and other organisms
☒	☐ Clinical data
☒	☐ Dual use research of concern
☒	☐ Plants

Methods

n/a	Involved in the study
☒	☐ ChIP-seq
☒	☐ Flow cytometry
☒	☐ MRI-based neuroimaging

Antibodies

Antibodies used

The following antibodies were used in this study:

Anti-ABCB4, clone P3II-26 - Enzo Life Sciences (Villeurbanne, France) - Ref ALX-801-028
Anti-ABCC2, clone M2I4 - Enzo Life Sciences (Villeurbanne, France) - Ref ALX-801-015-C250
Anti-α-tubulin, clone 1E4C11 - ProteinTech (Manchester, UK) - Ref 66031-1-Ig

Validation

The validation of each antibody was performed by state-of-the-art methods and include the specificity of signals (immunoblots and immunofluorescence) in cells expressing the antigen or not, as well as the subcellular localization of their staining (immunofluorescence).

Eukaryotic cell lines

Policy information about cell lines and Sex and Gender in Research

Cell line source(s)

The following cells were used in this study:

Human Embryonic Kidney 293 (HEK293) - ATCC - Ref CRL-1573
Human hepatocellular carcinoma (HepG2) - ATCC - Ref HB-8065

Authentication

The cells used in this study did not require further authentication beyond the certificates provided by the supplier (ATCC).

Mycoplasma contamination

Cell lines were regularly tested for the absence of mycoplasma, using a commercial kit (MycoAlert mycoplasma detection kit - Lonza).

Commonly misidentified lines
(See ICLAC register)

No misidentified lines were used in this study.

Plants

Seed stocks

Not applicable

Novel plant genotypes

Not applicable

Authentication

Not applicable
